# Sensitisation of cancer cells to radiotherapy by serine and glycine starvation

**DOI:** 10.1038/s41416-022-01965-6

**Published:** 2022-09-17

**Authors:** Mattia Falcone, Alejandro Huerta Uribe, Vasileios Papalazarou, Alice C. Newman, Dimitris Athineos, Katrina Stevenson, Charles-Etienne Gabriel Sauvé, Yajing Gao, Jin K. Kim, Michael Del Latto, Maria Kierstead, Chao Wu, J. Joshua Smith, Paul B. Romesser, Anthony J. Chalmers, Karen Blyth, Oliver D. K. Maddocks

**Affiliations:** 1 School of Cancer Sciences, Wolfson Wohl Cancer Research Centre, University of Glasgow, Glasgow, UK; 2 Cancer Research UK Beatson Institute, Glasgow, UK; 3 Colorectal Service, Department of Surgery, Memorial Sloan Kettering Cancer Center, New York, NY USA; 4 Human Oncology and Pathogenesis Program, Memorial Sloan Kettering Cancer Center, New York, NY 10065 USA; 5 Department of Radiation Oncology, Memorial Sloan Kettering Cancer Center, New York, NY 10065 USA; 6 Early Drug Development Service, Department of Medicine, Memorial Sloan Kettering Cancer Center, New York, NY 10065 USA; 7 Present Address: Division of Oncogenomics, The Netherlands Cancer Institute, Plesmanlaan 121, 1066 CX Amsterdam, The Netherlands

**Keywords:** Metabolomics, Cancer metabolism

## Abstract

**Background:**

Cellular metabolism is an integral component of cellular adaptation to stress, playing a pivotal role in the resistance of cancer cells to various treatment modalities, including radiotherapy. In response to radiotherapy, cancer cells engage antioxidant and DNA repair mechanisms which mitigate and remove DNA damage, facilitating cancer cell survival. Given the reliance of these resistance mechanisms on amino acid metabolism, we hypothesised that controlling the exogenous availability of the non-essential amino acids serine and glycine would radiosensitise cancer cells.

**Methods:**

We exposed colorectal, breast and pancreatic cancer cell lines/organoids to radiation in vitro and in vivo in the presence and absence of exogenous serine and glycine. We performed phenotypic assays for DNA damage, cell cycle, ROS levels and cell death, combined with a high-resolution untargeted LCMS metabolomics and RNA-Seq.

**Results:**

Serine and glycine restriction sensitised a range of cancer cell lines, patient-derived organoids and syngeneic mouse tumour models to radiotherapy. Comprehensive metabolomic and transcriptomic analysis of central carbon metabolism revealed that amino acid restriction impacted not only antioxidant response and nucleotide synthesis but had a marked inhibitory effect on the TCA cycle.

**Conclusion:**

Dietary restriction of serine and glycine is a viable radio-sensitisation strategy in cancer.

## Introduction

Radiotherapy is a common treatment modality used across many types of cancer and is used in ~50% of all cancer patients [1] as part of disease treatment/management. While radiotherapy provides important clinical benefits, for large numbers of patients, many tumours recur after treatment because of innate or acquired radioresistance. The ability of radiotherapy to eliminate tumours or impede their growth is achieved primarily through the induction of DNA damage [2]. While this damage can occur through direct action on DNA, the majority (~70%) occurs indirectly and is mediated by radiation-induced reactive oxygen species (ROS) [3]. Cancer cells mitigate the effects of radiotherapy by engaging antioxidant responses to neutralise ROS, and by repairing damaged DNA. These defence mechanisms, which prevent cancer cell death and directly promote radioresistance, are entirely dependent on metabolic processes: the synthesis of nucleotides to repair DNA and the synthesis/turnover of the antioxidant glutathione (GSH) require glucose and amino acid metabolism.

Research groups are increasingly using ‘multi-omics’ systems biology approaches, including metabolomics, to understand how cancer cells respond to radiation [4]. While DNA repair and redox-related pathways are consistently identified as major response pathways when cells are irradiated in culture, it is clear that the metabolic response to radiation is wide-ranging and complex, influencing central carbon and lipid metabolism in numerous ways [5, 6]. In contrast to these broad multi-omics studies have been more targeted, clinically inspired studies using small molecules with metabolic activity to improve the efficacy of radiotherapy [7–13]. The most common approach has been to target mitochondrial respiration using molecules such as biguanides (complex I inhibition) and atovaquone (complex III inhibition), prompting multiple clinical trials. The radio-sensitising effect of mitochondrial inhibitors is largely attributed to diminishing oxygen consumption which in turn decreases hypoxia, leaving more abundant oxygen for radiation-induced free radical generation and DNA damage [14]. Beyond these effects on oxygen levels, TCA cycle inhibition is predicted to cause radio-sensitisation by additional mechanisms, for example inhibiting the mitochondrial enzyme dihydroorotate dehydrogenase (DHODH) which is required for pyrimidine synthesis [14]. Furthermore, it has been revealed that inhibiting mitochondrial nutrient transport via mitochondrial citrate (SLC25A1) and dicarboxylate (SLC25A10) transporters can also promote radiation sensitivity [15, 16]. While this work points to a role for mitochondrial-cytoplasmic nutrient exchange to control radiation-induced redox response, detailed metabolic analysis is lacking.

While targeted small-molecule approaches show promise as metabolic radio-sensitisation strategies, a potential limitation is the issue of adaptation by metabolic rewiring [17]. Given the great complexity of the metabolic network it is inevitable that precise targeting of metabolism can be undone by changes in reaction direction and flux. An alternative (and complementary) approach to controlling cancer cell metabolism is to modulate nutrient availability. One advantage of this approach is the potential to simultaneously disrupt multiple metabolic pathways involved in treatment resistance. Both methionine and arginine restriction have long histories of exploration as anticancer strategies, either by dietary control or enzymatic mediated depletion, (reviewed in refs. [18, 19]), and both have been found to cause radio-sensitisation [20–23].

Dietary restriction of the non-essential amino acids serine and glycine has been shown to slow tumour growth and improve survival in several preclinical models of cancer, including genetically engineered mouse models (GEMMs), allografts and xenografts [24, 25]. Rational combination of serine and glycine restriction with small-molecule metabolic inhibitors and chemotherapies has shown additive effects [24–29]. As serine and glycine are essential precursors for the synthesis of nucleotides and GSH, there is a compelling case for combining serine and glycine restriction with radiotherapy, but to date this combination has not been investigated. Here we show that serine and glycine restriction can radiosensitise a range of cancer cells in vitro and in vivo, by simultaneously impacting multiple radiation-responsive metabolic pathways.

## Results

### Serine and glycine restriction sensitises cancer cells to radiation in vitro

**Fig. 1:**
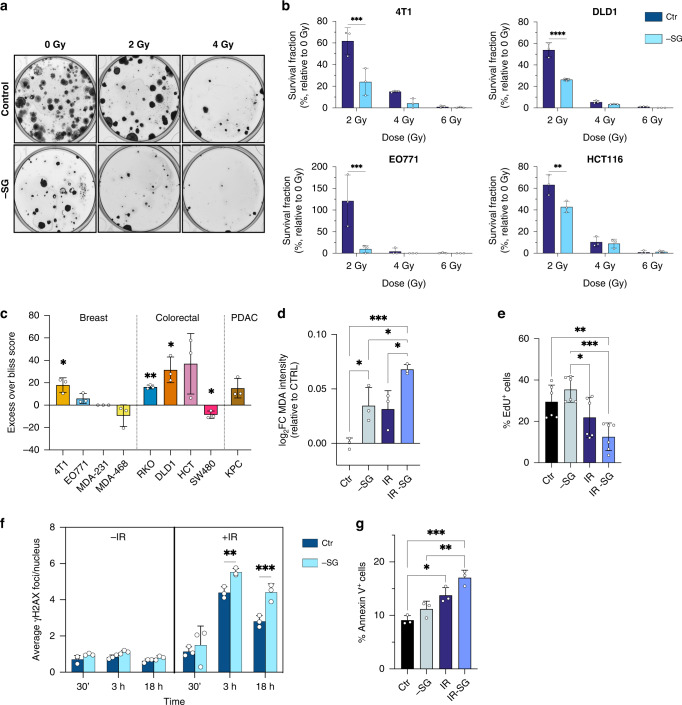
Serine and glycine starvation sensitises cancer cells to radiation in vitro. **a** Representative image of a clonogenic assay of 4T1 cells (as quantified in **b**) cultured with medium containing serine and glycine (Control) or not (-SG) and irradiated with increasing doses of X-ray radiation. **b** Quantification of clonogenic assays as surviving fraction relative to non-irradiated controls (mean +/− SD, *n* = 3 experiments in biological triplicate for each cell line, Two-way ANOVA with Šídák’s multiple-comparisons test, *P < 0.05, **P < 0.005, ***P < 0.0005, ****P < 0.00005). **c** Excess over Bliss score based on clonogenic assays of displayed lines cultured with media containing serine and glycine or not and irradiated with a final dose of 2 Gy X-ray radiation (mean +/−SD, *n* = 3 experiments in biological triplicate for 4T1 and one experiment in biological triplicate for other cell lines, One-sample *t* test, *P < 0.05, **P < 0.005, ***P < 0.0005, ****P < 0.00005). **d** Quantification of malondialdehyde (MDA) adduct intensity relative to Ctr measured using immunofluorescence staining 72 h after treatment. 4T1 cells were cultured with media containing serine and glycine (Ctr) or not (-SG) and irradiated with a final dose of 4 Gy X-ray radiation (IR) (mean +/−SD, one experiment in biological triplicate, One-way ANOVA with Tukey’s multiple-comparisons test, *P < 0.05, **P < 0.005, ***P < 0.0005, ****P < 0.00005). **e** Quantification of EdU incorporation measured using flow cytometry 48 h after treatment. 4T1 cells were cultured with media containing serine and glycine (Ctr) or not (-SG) and irradiated with a final dose of 4 Gy X-ray radiation (IR). Numbers indicate percentage of EdU positive cells (mean +/−SD, *n* = 2 experiments in biological triplicate, One-way ANOVA with Tukey’s multiple-comparisons test, *P < 0.05, **P < 0.005, ***P < 0.0005, ****P < 0.00005). **f** Quantification of γ-H2AX foci at different time points using immunofluorescence staining in 4T1 cells cultured with media containing serine and glycine (Ctr) or not (-SG) and irradiated or not with a final dose of 4 Gy X-ray radiation (+/−IR) (mean +/−SD, one experiment in biological triplicate, Two-way ANOVA with Tukey’s multiple-comparisons test, *P < 0.05, **P < 0.005, ***P < 0.0005, ****P < 0.00005). **g** Quantification of Annexin-V-positive cells using flow cytometry 24 h after treatment. 4T1 cells cultured with media containing serine and glycine (Ctr) or not (-SG) and irradiated with a final dose of radiations of 4 Gy (IR). Numbers indicate percentage of positive cells (mean +/−SD, one experiment in biological triplicate, One-way ANOVA with Tukey’s multiple-comparisons test, *P < 0.05, **P < 0.005, ***P < 0.0005, ****P < 0.00005).

To test the impact of serine and glycine (SG) restriction on the response of cancer cells to radiotherapy, we irradiated a range of cancer cell lines – including murine breast cancer lines (4T1 and EO771), human breast cancer lines (MDA-MB-231, MDA-MB-468), human colorectal cancer lines (HCT116, DLD1, SW480) and a murine pancreatic cancer line (KPC) in the presence or absence of SG. In vitro clonogenicity, the gold standard measure of radiosensitivity, was used to evaluate response. In the majority of cell lines, SG restriction augmented radiation-associated reductions in surviving fraction, indicating radio-sensitisation (Fig. 1a, b and Supplementary Fig. S1a). MDA-MB-468 and SW480 cells, which are known to be highly resistant to the impact of serine and glycine restriction [30], were not sensitised to radiotherapy. To evaluate the degree of synergy between SG starvation and radiation, we calculated the excess over Bliss score [31, 32] using doses of 2/4/6 Gy radiation. Several lines, including 4T1, showed positive scores, indicating a synergistic effect, whereas the SG starvation-resistant cell lines SW480 and MDA-MB-468 were negative (Fig. 1c and Supplementary Fig.  S1b). As MDA-MB-231 were so highly sensitive to SG starvation (causing a complete absence of colonies), there was no possibility that the addition of radiation could further damage the cells, meaning a synergy score could not be calculated. We also noted that as radiation dose increased, synergy generally decreased, an expected trend as when the radiation dose is more lethal there is less scope for the added benefit of starvation (Supplementary Fig. S1b).

### Serine and glycine starvation increase radiation-induced damage in vitro

To further assess the impact of SG starvation on cellular response to radiation we analysed markers of proliferation (EdU incorporation) cell death (Annexin V) both by FACS, and ROS (malondialdehyde/MDA) and DNA damage (gamma-H2AX foci), both by quantitative immunocytochemistry using an automated high content confocal image analysis platform (Perkin Elmer Opera). Whereas colony formation assays used above to assess radio-sensitisation require very low cell numbers (e.g., 200–500 cells seeded per well allowed to grow for weeks) for subsequent assays, we used higher seeding densities (to provide enough material for analysis) and shorter time courses to assess responses. As cells are more resistant to stress when grown at high versus low density [33, 34], cells tolerated doses of 4–10 Gy more readily than in colony formation assays, providing ample adherent cells for analysis. In the majority of cell lines, we observed an expected increase in ROS exposure in response to radiation, which showed a trend to be further increased by SG starvation (Fig. 1d and Supplementary Fig. S1c, d). The percentage of cells in the S-phase, indicated by EdU incorporation, tended to be approximately 50% lower in irradiated-SG starved cells versus irradiated only (Fig. 1e and Supplementary Fig. S2a). We noted that whereas HCT116 cells (p53 wild-type) showed decreased S-phase and evidence of G_1_ cell cycle arrest in response to SG starvation only (Supplementary Fig. S2a), 4T1 cells (p53 null [35]) did not show diminished S-phase. These observations correlate with previous work on the role of p53 in the response to SG starvation, in which p53 wild-type cells undergo G_1_ arrest whereases p53 null cells accumulate in G_2_ [24].

Nuclear DNA-damage foci, identified by γH2AX staining, a dynamic marker of radiotherapy-induced DNA damage [36], were rapidly induced by radiation, as expected, and in four out of six cell lines the number of foci were further increased by combination with SG starvation (Fig. 1f and Supplementary Fig. S2b, c). Interestingly, in the case of MDA-MB-231 cells, SG starvation alone increased the number of DNA-damage foci but did not further increase foci when combined with radiation beyond 3 h. In all four cell lines tested, radiation alone showed a consistent ability to increase apoptosis, as indicated by cell surface phosphatidylserine expression detected by annexin-V binding (Fig. 1g and Supplementary Fig. S2d). SG starvation alone had a smaller impact on apoptosis but showed a trend to further increase apoptosis in combination with radiation in three of four cell lines tested. Taken together, these results indicate that SG starvation can increase radiation-induced damage and sensitise a range of different cancer cell lines to radiation in vitro.

### Radiation has wide-ranging effects on the cellular metabolome

**Fig. 2:**
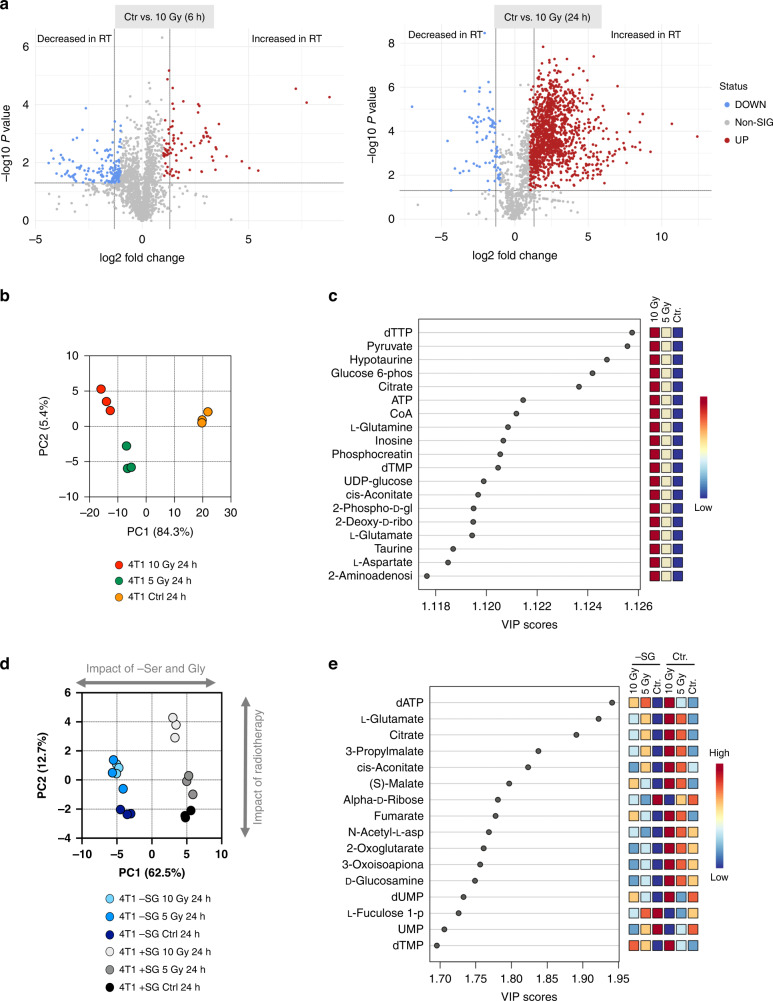
Radiotherapy has a profound impact on the cellular metabolome, which is shifted by serine and glycine starvation. **a** Volcano plots showing differential metabolite abundance for the experiment described in **a**. Control and 10 Gy conditions were compared, and differentially significant metabolites are highlighted. The fold-change threshold was set to 2.0 and the *P* value to 0.05 (raw) with an equal group variance. **b** Spatial division displayed as PCA plot based on the metabolomic profile of 4T1 cells cultured in complete medium irradiated with increasing dose of X-ray radiation (0/Ctr, 5 and 10 Gy) and harvested at 24 h post radiation (one experiment in biological triplicate). **c** Key metabolites separating the culture conditions based on variable importance in projection (VIP) in PLS-DA analysis of the metabolomic profile from the experiment described in Fig. 2a. **d** Spatial division displayed as PCA plot based on the metabolomic profile of 4T1 cells cultured with media containing serine and glycine (Ctr) or not (-SG) and irradiated with increasing doses of X-ray radiation (0, 5 and 10 Gy) for 24 h (mean +/− SD, one experiment in biological triplicate). **e** Key metabolites separating the culture conditions based on variable importance in projection (VIP) in PLS-DA analysis of the metabolomic profile from the experiment described in 2d.

To assess the general metabolic impact of radiotherapy on cancer cells, we exposed 4T1 cells to single doses of 5 Gy and 10 Gy X-ray radiation in vitro and analysed samples by liquid chromatography-mass spectrometry (LCMS) using a pHILIC column to yield a broad analysis of metabolites in central carbon metabolism. In similarity to the ROS/DNA-damage/apoptosis assays described above, LCMS experiments were seeded at a higher density than colony formation experiments to provide enough material for analysis. Cell counts illustrate that under these conditions, 5–10 Gy radiation impeded cell growth but provided ample adherent cells for LCMS analysis (Supplementary Fig. S3a). Just 6 h after treatment, changes in the metabolome of the irradiated cells could be detected using supervised analysis (partial least squares discriminate analysis, PLS-DA volcano plot) (Fig. 2a) and by principal component analysis (PCA) (Supplementary Fig. S3b). After 24 h the metabolomic differences became even more marked and were seen to be dose-dependent (Fig. 2a, b). Volcano plots of non-irradiated control versus 10 Gy irradiated cells show 89 metabolites increased and 169 decreased after 6 h, however, by 24 h over 1300 metabolites are increased compared to only 77 decreased (Fig. 2a). We speculate that the large number of increased metabolites is likely to be a feature of both specific, adaptive changes and more passive metabolite accumulation resulting from slowed cell growth.

Analysis of specific metabolites changed in response to radiation alone revealed hits from key energetic and anabolic pathways: nucleotides and related metabolites (dTTP, dTMP, ATP, inosine, 2-deoxyribose) were very strongly represented, reflecting radiation-induced DNA damage and repair. Glycolysis (glucose 6-phosphate, pyruvate), and the TCA cycle (citrate, CoA, cis-aconitate) were also highlighted, including TCA cycle-related amino acids (aspartate, glutamate, glutamine) (Fig. 2c). In all cases, the pathways identified as ‘most changed’ showed increased metabolite levels versus controls.

### Serine and glycine starvation shifts the metabolic response to radiotherapy in multiple pathways

Next, we analysed the metabolomic response to SG starvation and radiation combined, performing a large set of experiments exposing nine cancer cell lines to 5 Gy and 10 Gy radiation in the presence or absence of exogenous serine and glycine. Starting with 4T1 cells, PCA revealed that SG starvation had a dramatic impact on the metabolome, with starved cells very clearly separated from control cells by this unsupervised analysis (Fig. 2d). While the impact of radiation was clear and substantial (as already seen in Fig. 2a, b), SG starvation was the dominant driver of metabolic change when combined with radiation, i.e., PC1 (62.5% variance), driven by SG starvation response, was greater than PC2 (12.7% variance) driven by radiation response (Fig. 2d).

PLS-DA analysis of irradiated control and SG starved cells revealed a shift towards greater representation of TCA cycle metabolites (citrate, cis-aconitate, malate, fumarate, 2-oxoglutarate), with the continued representation of nucleotides (dATP, dUMP, dTMP), but lack of glycolytic intermediates (Fig. 2e). Intriguingly we observed that for all of the most altered TCA cycle metabolites, SG starvation strongly diminished the radiation-induced metabolite increases seen under fed conditions (Fig. 2e). This unexpected observation was replicated across the most SG starvation-sensitive cell lines (e.g., MDA-MB-231 and RKO cells), but was not seen in SG starvation-resistant cell lines, such as SW480 and MDA-MB-468 cells) (Supplementary Fig. S3c). As well as influencing the TCA cycle, the switch to SG-free medium also caused suppression of GSH levels. Whereas cells responded to radiation with increased GSH levels under control conditions, under SG starvation (with or without radiation) GSH levels were lower than in the non-starved equivalents (Supplementary Fig. S3c). This is a logical observation given GSH requires glycine (and glutamate and cysteine) for de novo synthesis, and we have previously shown that SG starvation lowers GSH and increases ROS levels in cancer cells [24]. This observation also correlates with the trend for increased ROS damage seen during combined SG starvation and radiation (Fig. 1d and Supplementary Fig. S1c, d).

**Fig. 3:**
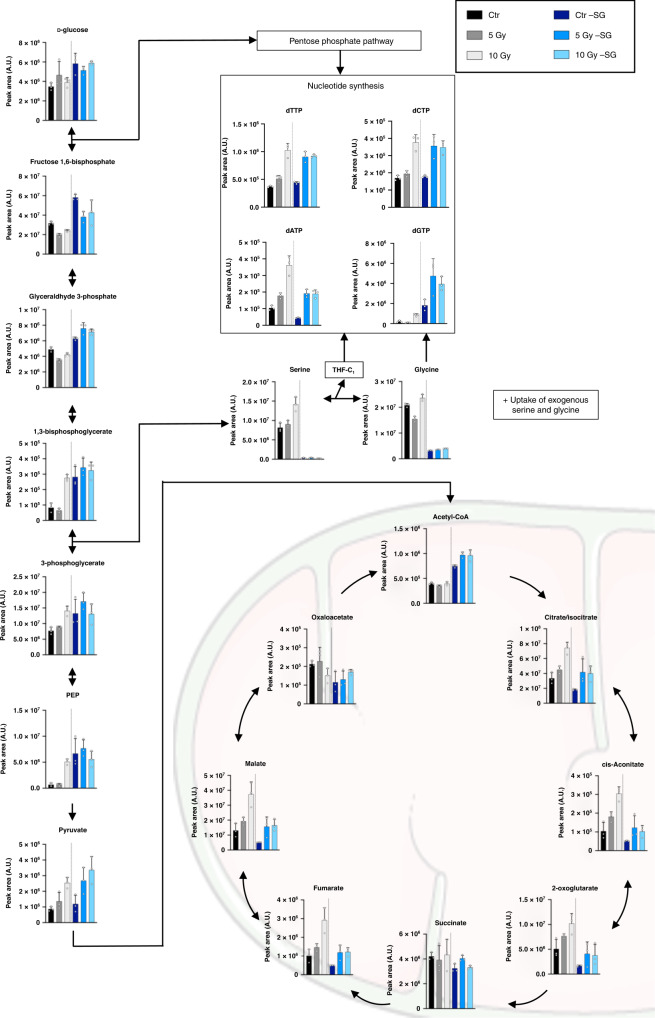
Impact of radiotherapy on glycolysis, TCA cycle and nucleotides. Metabolite levels analysed by LCMS in 4T1 cells cultured with media containing serine and glycine (Ctr) or not (-SG) and irradiated with increasing doses of X-ray radiation (0, 5 and 10 Gy) for 24 h (mean +/−SD, one experiment in biological triplicate). See Table S1 for statistical comparisons.

Analysis of individual metabolites in these altered pathways emphasises the diminution by SG starvation of radiation-induced increases in TCA cycle metabolites, dATP, GSH and NADPH. (Fig. 3 and Supplementary Fig. S4). The impact of radiation and SG starvation on redox/ROS-related metabolites was dramatic. Interestingly, radiation alone caused a dose-dependent increase in GSH levels by 24 h, but only a comparatively modest increase in GSSG (oxidised glutathione) (Supplementary Fig. S4). This suggests that de novo GSH synthesis is activated by radiation and outpaces the demand for oxidation of GSH to GSSG by radiation-induced ROS. I.e. by increasing the total GSH pool, cells prevent the GSSG/GSH ratio increasing, despite the inevitable increase in GSSG caused by radiation-induced ROS (Supplementary Fig. S4). Paralleling the increase in GSH levels caused by radiation in fed conditions was a large elevation in NADPH and decrease in the NADP+/NADPH ratio (Supplementary Fig. S4). This change also reflects the increased antioxidant capacity (GSH) stimulated by radiation, as NADPH is the redox co-factor used to reduce GSSG to the antioxidant GSH. It was notable that while radiation increased both NAD+ and NADH levels, unlike the other redox-related metabolites, their ratio was much less altered, and they were relatively unaffected by SG starvation. This observation is likely to reflect the role of GSH and NADPH in the antioxidant response, a role not generally shared by NADH.

### RNA sequencing and pathway impact analysis show the dominant metabolic impact of serine and glycine starvation on radiation response

To further characterise the impact of SG starvation on the cellular response to radiotherapy, we performed RNA sequencing (RNA-Seq) on SG starved/irradiated 4T1 cells. The rationale for including transcriptomic in addition to metabolomic analysis was to provide a more complete biological picture of the response to radiotherapy/starvation. Metabolite fluxes can be highly dynamic (metabolites are interconverted in seconds) and changes in metabolite levels can be the result of multiple possible pathway changes. For example, observing a decrease in the level of a metabolite can be the result of increased degradation, increased utilisation (e.g., in an anabolic reaction), reduced production, or a combination of these, making interpretation of changed metabolite levels potentially ambiguous. Adding data on gene expression, in which directional changes of increased or decreased expression can generally be more directly interpreted in terms of pathway activity changes, makes an integrated analysis more powerful/reliable than metabolomics alone.

**Fig. 4:**
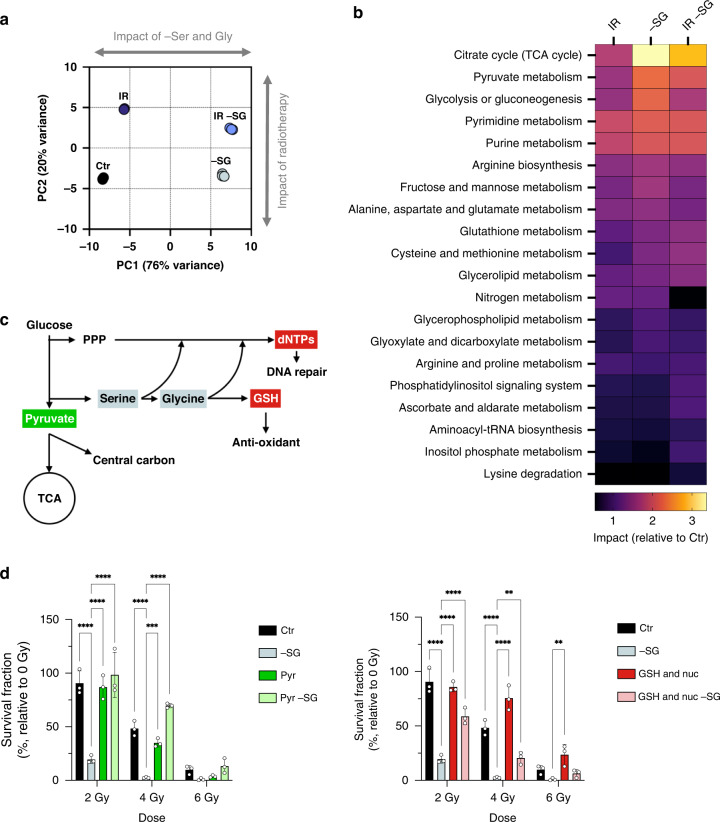
Integrated metabolomic-transcriptomic analysis and metabolite rescue. **a** Spatial division displayed as PCA plot based on RNA-Seq profile of 4T1 cells cultured with media containing either serine and glycine or not (-SG) and irradiated with a final dose of X-ray radiation 5 Gy (IR) for 24 h (one experiment in biological triplicate). **b** Joint pathway analysis based on both metabolomics (Fig. 2d) and gene expression (Fig. 4a) profiles of 4T1 cells cultured with media containing either serine and glycine (+SG) or not (-SG) and irradiated with a final dose of radiations 5 Gy (IR) for 24 h (one experiment in biological triplicate for both metabolomics profiling and RNA-Sequencing). **c** Schematic depicting serine/glycine biosynthesis and related pathways, and corresponding metabolites used for rescue experiments. **d** Quantification of clonogenic assays as surviving fraction relative to non-irradiated controls of 4T1 cells cultured with media containing serine and glycine (Ctr) or not (-SG), irradiated with increasing doses of X-ray radiation (0–6 Gy) and with or without supplementation with 2 mM Pyruvate or 2.5 mM reduced glutathione and nucleosides mix (GSH & Nuc) (mean +/−SD, one experiment in biological triplicate, Two-way ANOVA with Dunnet’s multiple-comparisons test, *P < 0.05, **P < 0.005, ***P < 0.0005, ****P < 0.00005).

Unsupervised principal component analysis of RNA-Seq data demonstrated very clear segregation of the experimental groups based on the transcriptional response to radiation/starvation (Fig. 4a). Interestingly, and in similarity to metabolomic data (Fig. 2d), the dominant driver of group separation was SG starvation (PC1, 76% variance) rather than radiation (PC2, 20% variance) (Fig. 4a). More detailed pathway-based analysis of RNA-Seq data (Supplementary Fig. S5a, b) emphasises the suppression of the TCA cycle by starvation, along with activation of ATF4 and serine synthesis pathways, as noted previously [37–39]. In addition, there was an expected impact of SG starvation on NRF2 and oxidative stress gene expression [26, 40] interestingly, we saw that SG starvation alone had a more profound impact on these pathways than radiation alone, and that combined treatment tended to reflect starvation-induced rather than radiation-induced changes (Supplementary Fig. S5a, b).

Next, we used an integrated ‘pathway impact analysis’ of the combined RNA-Seq and LCMS metabolomics data as a powerful method to globally assess response to SG starvation/radiation. The pathways most impacted by SG starvation alone in 4T1 cells were the TCA cycle, pyruvate metabolism, glycolysis and nucleotide metabolism, followed by a range of amino acids, fructose/mannose and glutathione (Fig. 4b). These pathways were also impacted, but to a lesser extent, by radiation alone. Notably, the most impacted pathways in combined SG starvation and radiotherapy treatment mirrored those seen for SG starvation alone, with the exception of glycolysis, validating the dominant impact of starvation seen in the PCA analysis.

Given the major impact seen on the TCA cycle, we plotted a schematic representation of individual metabolites and metabolic enzymes in this pathway, indicating the direction of change in response to individual and combined radiation/SG starvation (Supplementary Fig. S5c). Under control (fully fed) conditions, radiotherapy was on average shown to increase metabolite levels and enzyme expression in the TCA cycle. In contrast, compared to control conditions, SG starvation alone tended to uniformly suppress the TCA cycle. When both radiation and SG starvation were applied, the suppressive effect of SG starvation was totally dominant, preventing radiation-induced upregulation.

### Supplementary pyruvate, nucleotides and antioxidants rescue the radio-sensitising effect of SG starvation

To assess the relative importance of individual metabolites identified as involved in combined SG starvation and radiation response (Fig. 4c), we conducted metabolite rescue experiments. Remarkably, supplementary pyruvate alone produced complete/nearly complete rescue of the radio-sensitising effects of SG starvation in 4T1, DLD1 and KPC cells but had very little impact on highly SG starvation-sensitive cell lines MDA-MB-231 and RKO (Fig. 4d and Supplementary Fig. S6a). Combined GSH and nucleoside supplementation produced rescue in the majority of cell lines and was better than pyruvate in several lines, including MDA-MB-231 and HCT116. As expected, supplementary pyruvate, GSH and nucleoside have less impact on radiation response in SG replete conditions (Fig. 4d and Supplementary Fig. S6b). We have previously confirmed that exogenous GSH can enter cancer cells and provide an antioxidant effect [24, 34]. We confirmed that exogenous pyruvate augmented the TCA cycle in starved/irradiated cells (Supplementary Fig. S6c). The impact of supplementary antioxidants-only on rescue was generally mild, emphasising that SG starvation and radiation impact multiple metabolic pathways, not just those related to ROS (Supplementary Fig. S6d, e). Overall, these results underline the multi-functional protective roles that exogenous serine and glycine play in the response of cancer cells to radiation via multiple metabolic pathways that are simultaneously impacted by radiation.

### Serine and glycine restriction sensitises in vivo tumours and patient-derived tumour organoids to radiotherapy

**Fig. 5:**
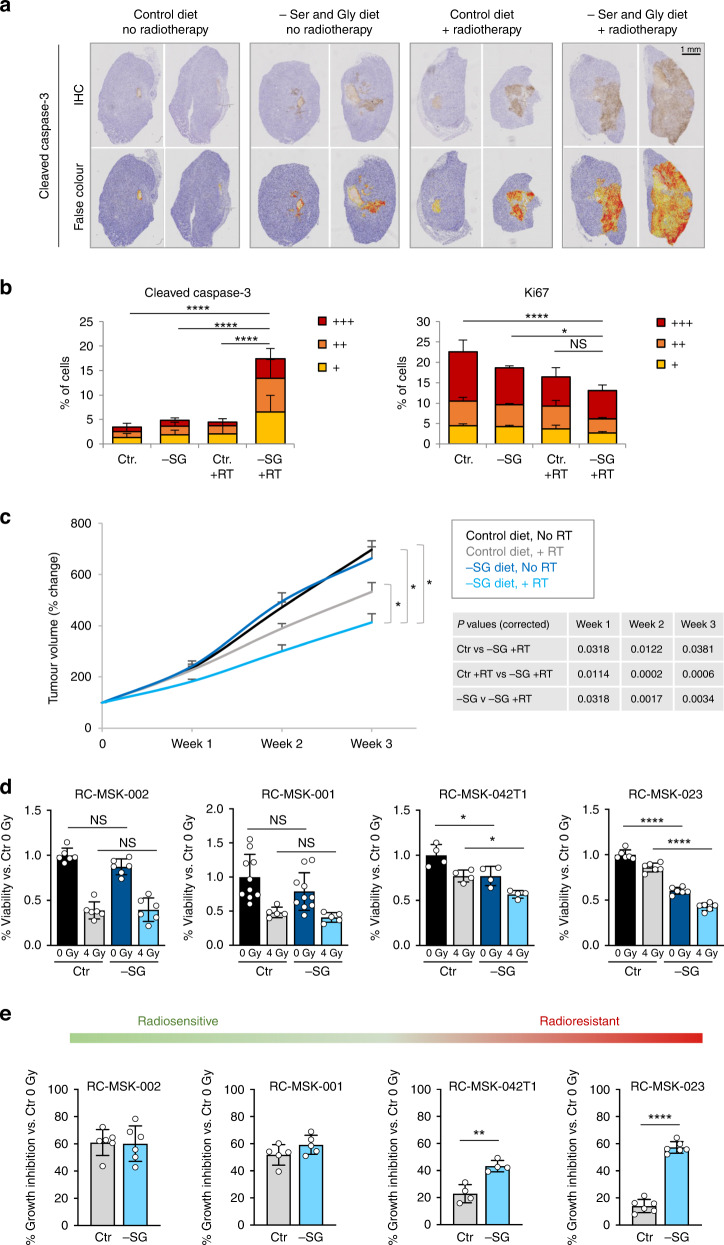
Serine and glycine starvation sensitises tumours and tumour organoids to radiotherapy. **a** Representative formalin fixed paraffin embedded tissue sections from KPC cell syngeneic subcutaneous isografts were stained for cleaved caspase-3 protein. Once measurable tumours had formed, mice were transferred to a diet regime consisting of either a control diet containing serine and glycine (Control diet) or a matched diet lacking serine and glycine (-SG diet); after 3 days on diet, tumours were treated with 20 Gy targeted X-ray radiation. None of the tumours reached the size endpoint due to skin ulceration occurring in all experimental groups, so all tumours were harvested for IHC analysis within 7 days of radiation. **b** Halo image analysis software was used to quantify IHC slides of cleaved caspase-3- and Ki67-stained cells (mean +/− SEM, *n* = 4 mice per condition, *n* = 2 tumours per mouse, two-way ANOVA with Tukey’s multiple-comparisons test, *P < 0.05, **P < 0.005, ***P < 0.0005, ****P < 0.00005). Per cell staining intensity values and cleaved caspase-3 and Ki67 are defined as positive (+), strongly positive (++) or very strongly positive (+++) versus controls. **c** 4T1 cells were injected into the mammary fat pad of Balb/C mice to form syngeneic orthotopic tumours. Once isograft tumours had formed, mice were transferred to a diet regime consisting of either a control diet containing serine and glycine (Control diet) or a matched diet lacking serine and glycine (-SG diet); after 3 days on diet, tumours were treated with 15 Gy targeted X-ray radiation. Data are averages of 2–3 weekly tumour measurements, bars are SEM (Control diet No RT *n* = 13 mice, Control diet +RT *n* = 12 mice, -SG diet No RT *n* = 15 mice, -SG diet +RT *n* = 12 mice). Tumour volume is plotted from the time of diet change. Statistical comparisons were made using two-sided unpaired *t* test with Holm–Sidak’s *P* value correction for multiple comparisons, *P < 0.05, **P < 0.005, ***P < 0.0005, ****P < 0.00005. **d** Four patient-derived rectal cancer tumour organoid lines were grown in the presence (Ctr) or absence (-SG) of serine and glycine. After 48 h, organoids were irradiated with a dose of 4 Gy and allowed to grow for a further 6 days. At endpoint organoids were quantified using Cell Titre Glo reagent. Data is mean +/− SD for each organoid in one experiment with at least four biological replicates (individual replicates are shown, two-way ANOVA with Tukey’s multiple-comparisons test, *P < 0.05, **P < 0.005, ***P < 0.0005, ****P < 0.00005). **e** Data shown in **d** calculated as % growth inhibition caused by 4 Gy radiation versus Control (Ctr) untreated (0 Gy) condition. Data is mean +/− SD for each organoid in one experiment with at least four biological replicates (individual replicates are shown, Unpaired *t* test two-tailed *P < 0.05, **P < 0.005, ***P < 0.0005, ****P < 0.00005).

To test the ability of SG starvation to sensitise tumours to radiation in vivo, we used two syngeneic mouse models of cancer. Initially, we utilised a syngeneic allograft model of pancreatic cancer where C57Bl6/J mice were subcutaneously transplanted with KPC cells (derived from *Pdx1-cre;KrasG12D/+;Trp53R172H/+* mice [41, 42]) and therapeutically treated with a single 20 Gy dose of tumour-targeted radiation with or without a SG deficient diet. Due to the rapid growth and invasive nature of the KPC cells, we found that several tumours (in all experimental groups) began to cause skin ulceration (a clinical endpoint) and decided to terminate the experiment and collect all tumour samples within 7 days of radiation. Tumour cross sections were stained by immunohistochemistry for the apoptosis marker cleaved caspase-3 and the proliferation marker Ki67. While radiation alone, or starvation alone, showed some evidence of increased apoptosis and decreased proliferation, the combination of SG deficient diet and radiotherapy showed the greatest response (Fig. 5a, b and Supplementary Fig. S7a). At this short temporal endpoint, tumour volume was not significantly different between groups, as expected (Supplementary Fig. S7b). Next, we employed a syngeneic orthotopic breast cancer model transplanting 4T1 murine mammary cancer cells into the mammary fat pad of wild-type Balb/c mice, feeding a control or SG deficient diet with or without a single 15 Gy dose of tumour-targeted radiation. The combination of SG starvation and radiotherapy showed significantly greater inhibition of tumour growth than either intervention alone (Fig. 5c and Supplementary Fig. S7c).

To further test the clinical relevance of our observations, we used rectal cancer patient-derived organoids (PDOs) maintained as part of a prospective biorepository at MSK. Radiotherapy is an integral part of modern, first-line treatment in rectal cancer prior to surgery [43, 44]. While some rectal tumours are radiosensitive, many are radioresistant, and there is a clinical need to develop interventions that sensitise rectal tumours to radiotherapy. Radiosensitive RC-MSK-002 & RC-MSK-001 PDOs responded to 4 Gy radiation with 50–60% growth inhibition in control conditions, which was not further increased by SG starvation (Fig. 5d, e). In contrast, the radioresistant RC-MSK-042T1 and RC-MSK-023 PDOs showed only 15–20% growth inhibition in response to 4 Gy radiation under control conditions, which was increased to 40–60% growth inhibition (I.e. comparable with radiosensitive PDOs) under SG starvation (Fig. 5d, e). Taken together, these results indicate that dietary SG starvation can increase the sensitivity of tumours and rectal cancer PDOs to radiotherapy, with the potential to radiosensitise otherwise resistant cancer cells.

## Discussion

We have assessed the metabolic response of colorectal cancer, breast cancer and pancreatic cancer models to radiotherapy and identified the potential for radio-sensitisation with SG starvation. Radiotherapy is a key component of the multimodal treatment of breast and colorectal cancer; for example, ~40% of newly diagnosed rectal cancer patients (30% of the CRC population) are referred for radiotherapy prior to surgical resection. For newly diagnosed rectal cancer patients receiving pre-operative radiotherapy, there is a curative outcome (rendering surgery unnecessary) in 10–15% of patients, whereas ~20% show no improvement. We anticipate that interventions that potentiate radiotherapy could significantly improve the curative outcome in this population and diminish the number showing no response. The use of radiation in pancreatic cancer is more controversial. The anatomical location of the pancreas makes it difficult to deliver high doses of radiation without inducing significant toxicity in adjacent organs. Several studies have reported improved overall survival using conventional radiation therapy in locally advanced pancreatic cancer; however, other studies indicated no benefit or even decreased survival (reviewed by Hazard [45]). Fortunately, technological developments, including intensity modulated radiation therapy (IMRT) and stereotactic body radiation therapy (SBRT), have improved accuracy, enabling the sparing of normal tissues and dose escalation to tumours [46, 47]. Identifying ways to further sensitise cancer cells to radiotherapy could help translate SBRT to an intervention that increases overall survival and quality of life in patients with this cancer of unmet need.

Previous studies have demonstrated that radiotherapy has a range of metabolic impacts on cancer cells. Here we show that radiotherapy has strong impacts on pathways known to be involved in antioxidant and DNA-damage repair, i.e., glutathione synthesis and turnover, and nucleotide metabolism. Unexpectedly, we identified a profound impact of radiotherapy on the TCA cycle, a phenomenon replicated across nine cancer cell lines used in this study. While we had hypothesised that SG starvation would impede antioxidant and nucleotide metabolism responses, we were surprised to note the degree to which starvation also impacted the TCA cycle, particularly in those cell lines in which starvation had the greatest radio-sensitising effect. In previous work, we have shown that SG starvation can impact the TCA cycle [24], however, this effect was limited to a relatively short-term (3–6 h) transient increase in glucose-derived TCA cycle intermediates. In the present study, we saw that SG starvation in combination with radiation caused a decrease in steady-state TCA cycle metabolites that persisted for at least 24 h. This observation correlates with prior work demonstrating serine to be an allosteric activator of the glycolytic enzyme PKM2, and that conversion of phosphoenolpyruvate to pyruvate by PKM2 is inhibited under serine starvation [48]. This impediment to pyruvate synthesis is predicted to diminish levels of TCA cycle metabolites and explains why supplementary pyruvate was able to rescue cells from the radio-sensitising effects of SG starvation.

There is significant published preclinical data suggesting that metabolic interventions, particularly small-molecule inhibitors of mitochondrial metabolism, have potential as radio-sensitisation strategies. The most commonly hypothesised mechanism of action for this interaction is that TCA cycle/OXPHOS inhibition lowers oxygen consumption by tumour cells, increasing the local abundance of oxygen, elevating oxygen-derived ROS and increasing DNA damage [14]. The new data presented here suggest that an alternative/additional mechanism is that increased TCA cycle metabolism is a major component the metabolic response of cancer cells to radiotherapy. Prior work showing that inhibiting mitochondrial nutrient transport can induce radio-sensitisation [15, 16] supports this hypothesis.

We noted that the transition from in vitro assays with 4T1 cells to an orthoptic in vivo model generally showed a diminution of response to both SG starvation and radiotherapy, underlining the known differences between in vitro and in vivo cell growth/proliferation. Compared to the in vitro situation, where complete removal of exogenous amino acids is possible, significant depletion is achieved in vivo, but not complete removal [25]. To aid potential clinical translation, future preclinical work could seek to employ fractionated radiotherapy dosing in tumour models where a SG restricted diet is used.

While mitochondrial inhibitors have shown preclinical promise as radio-sensitisers, and more generally as potential anticancer agents, this promise has so far failed to be validated in clinical trials. A clear limitation of targeted small-molecule inhibitors of metabolism is the complexity and plasticity of the metabolic network. Metabolic adaptation/rewiring by cancer cells has been shown to abrogate the impact of inhibitors that target specific metabolic enzymes known to drive anabolic reactions in tumours [17]. One solution to this metabolic plasticity is to use alternative therapeutic approaches which target multiple metabolic pathways simultaneously but do so by removing the supply of precursors rather than complete pathway inhibition (which is more likely to cause toxicity). Dietary nutrient limitation is one such strategy that is showing preclinical promise in a wide range of cancer models. The data in this study suggest that dietary limitation of serine and glycine is a potentially efficacious strategy to sensitise a range of common cancers to radiotherapy.

## Methods

### Cell culture

Cell culture was performed, as described previously [49]. Unless otherwise stated, all cell lines used in this study were obtained from ATCC and cultured at 37 °C in 5% CO_2_ in a humidified incubator. 4T1 (*M. musculus*, female), E0771 (*M. musculus*, female), DLD1 (*H*. *sapiens*, male), cells were cultured in RPMI (Invitrogen, 31870025) supplemented with 10% FBS, 1% penicillin–streptomycin, 0.2% amphotericin B and glutamine (2 mM). MDA-MB-231 (*H*. *sapiens*, female), MDA-MB-468 (*H*. *sapiens*, female), RKO (*H*. *sapiens*) HCT116 (*H*. *sapiens*, male), SW480 (*H*. *sapiens*, male) and KPC (*M. musculus*) cell lines were cultured in DMEM (Invitrogen, 21969035) supplemented with 10% FBS, 1% penicillin–streptomycin, 0.2% amphotericin B and glutamine (2 mM). KPC lines were a gift from Jennifer Morton and Saadia Karim (Ximbio, 153474), and were isolated from the tumours of Pdx1-cre;LSL-Kras^G12D/+^;LSL-Trp53^R172H/+^ mice either with a mixed or pure C57BL/J background. Cell lines were tested for mycoplasma using Mycoalert (Lonza). Cell lines were authenticated by STR profiling using Promega GenePrint 10. A formulated medium lacking l-serine, and glycine was used as a base for creating experimental media. The 1×MEM (Gibco) was used as base medium supplemented with l-glutamine 2 mM, l-alanine 0.4 mM, l-proline 0.2 mM, l-glutamate 0.15 mM, l-aspartate 0.15 mM, l-asparagine 0.35 mM, vitamin mix (Sigma), 17 mM glucose, 10% dialysed FBS, 1% penicillin–streptomycin and 0.2% amphotericin B. The control experimental medium was the base medium supplemented with l-serine 0.4 mM and glycine 0.4 mM.

### In vitro radiation of cancer cells

Cultures were irradiated in tissue culture flasks at room temperature with 195 kV X-rays at a dose rate of 1.6 Gy/min in an Xstrahl RS225 cabinet.

### Clonogenic assay and rescue experiments

Cells were plated in triplicate in six-well plates overnight (seeding density: 4T1—200 cells, EO771—1700 cells, MDA-MB-231—2000 cells, MDA-MB-468—2000 cells, DLD1—500 cells, RKO—2000 cells, HCT116—2000 cells, SW480—1000 cells, KPC—1700 cells). Cells were washed with PBS and the relevant experimental media change was performed and followed by irradiation. Media were refreshed every 4 days, and after 12–14 days of culture, colonies were fixed and stained (0.05% (w/v) Crystal Violet (Millipore), 1% formaldehyde, 1% methanol in 1XPBS). Images were taken on a GelCount (Oxford Optronix), colonies were counted using ImgeJ and the surviving fraction was determined (SF = plating efficiency treated cells/plating efficiency Ctr; plating efficiency = #colonies/#cells plated). Alternatively, the area under the curve was calculated and used for statistical testing. For the rescue experiments the experimental media were supplemented with the following compounds as indicated in the figure legends: 2 mM pyruvate (GIBCO), 2.5 mM or 5 mM reduced glutathione (sigma), nucleosides mix (Sigma; 30 µM citidine, 30 µM guanosine, 30 µM uridine, 30 µM adenosine, 10 µM thymidine), 5 mM N-acetylcysteine (NAC, sigma), 2 uM ferrostatin-1 (Fer-1), 50 uM Trolox or 100 nM liproxstatin-1 (Lip-1).

### The excess over bliss score calculation

Based on the clonogenic assay results, excess over Bliss score [31, 32] was calculated and used to infer synergistic effect of serine/glycine depletion and radiotherapy. The score was calculated as following f_(IR+SG)_ – [(f_IR_ + f_SG_)-(f_IR_ x f_SG_)], were “f” indicates the single effect of either radiation or serine/glycine depletion (SG).

### Apoptosis analysis

In total, 300,000 cells were seeded into six-well plates in a complete medium and allowed to attach overnight. Cells were washed with PBS, and the relevant experimental media change was performed followed by irradiation. After 24 h, cells were harvested by enzymatic digestion, and an equal number of cells per sample were stained with an Annexin V/dead cell apoptosis kit according to the manufacturer’s procedure (Invitrogen, V35113). Data acquisition was performed by flow cytometry (FACS Verse BD Biosciences, San Jose, CA, USA) and analysis was done on FlowJo (v.10). The percentage of Annexin-V-positive cells was used for the analysis.

### Cell cycle analysis with EdU staining

In all, 250,000 cells were seeded into six-well plates in a complete medium and allowed to attach overnight. Cells were washed with PBS, and the relevant experimental media change was performed followed by irradiation. After 48 h, a pulse of 30 µM EdU was given for 45’. Then, cells were harvested by enzymatic digestion and an equal number of cells per sample were stained with a Click-iT^®^ Plus EdU Flow Cytometry Assay Kit according to the manufacturer procedure (Invitrogen, C10633). To measure DNA content, prior acquisition samples were treated with Ribonuease A (Quiagen, 20 mg/ml) and stained with propidium iodide (sigma, 1 µg/ml). Data acquisition was performed by flow cytometry (FACS Verse BD Biosciences, San Jose, CA, USA) and analysis was done on FlowJo (v.10).

### Immunofluorescence

In all, 5000 cells were seeded into 96-well plates (Greiner, flat bottom black polystyrene wells) in complete medium and allowed to attach overnight. Cells were washed with PBS, and the relevant experimental media change was performed followed by irradiation. At experimental endpoint, cells were washed with PBS and fixed for 15’ at 4 °C with 4% formaldehyde (sigma) and then washed with PBS. Cells were permeabilised (0.3% triton x 100 in PBS) at room temperature for 5’ twice, then cells were blocked (5% FCS, 0.5% BSA, 0.1% 100x ritonX in PBS) at 4 °C for 30’. Primary antibodies against PE-H2A.X Phospho (Ser^139^) (1/250; BioLegend, #613411), malondialdehyde (MDA) (1/600; Abcam, ab6463) were incubated in PBS 1%BSA 0.05% TritonX overnight at 4 °C. After, cells were washed three times in PBS 0.05% TritonX. For MDA staining, cells were then incubated for 3 h at 4 °C with secondary antibody (1:100; Alexa Fluor 568, Thermo scientific, A11011) and phalloidin (1:500; Alexa Fluor 488, Thermo Scientific, A12379), then cells were washed three times in PBS 0.05% TritonX. DNA was stained with DAPI (1:1000 DAPI (Thermo scientific, 62248) + 0.01%TritonX in PBS). The plates were then sealed and imaged using an Opera Phenix (Perkin Elmer) microscope platform with Harmony (Perkin Elmer) and analysed using Columbus software.

### Mice

All in vivo work was carried out in compliance with the Animals (Scientific Procedures) Act 1986 and the EU Directive 2010 (PPLs 70/8645 and PP6345023) and was sanctioned by the local ethical review process (University of Glasgow). *Mus musculus* cohorts were housed in a barrier facility proactive in environmental enrichment and maintained on a normal chow diet. For PDAC transplants: 2 × 10^6^ KPC cells were subcutaneously injected into the bilateral flanks of C57Bl6/J females aged 7–8 weeks at time of transplantation (Charles River, UK). KPC cells were derived from *Pdx1-cre;KrasG12D/+;Trp53R172H/+* mice [41]. Recipients were switched from normal chow to serine/glycine deficient or control diet 4 days after transplantation (at which point all mice had measurable tumours) in a manner ensuring consistent average starting tumour volume across the groups. After 3 days on experimental diets, tumours were irradiated as described below with 20 Gy radiation (mean tumour volume = 119 mm^3^ at time of radiation). For 4T1 transplants: 2.5 × 10^4^ cells were injected to the mammary fat pad of 8-week-old female Balb/C recipients (Charles River, UK) with appropriate anaesthesia and analgesia; and clips removed 7 days post-transplant. Recipients were switched to experimental diets when tumour volume = 150–230 mm^3^, in a manner ensuring consistent average starting volume across the groups. To ensure a consistent starting tumour volume, mice with tumour volume outside 150–230 mm^3^ were not enrolled in the study. After 3 days on experimental diets, tumours were irradiated as described below with 15 Gy radiation. All tumours were measured using callipers by technicians blinded to the aims of the study and the hypothesised outcome. Animals were humanely culled by Schedule 1 method and tissue processed for analysis. KPC and 4T1 cells were tested as mycoplasma-negative (as described above) prior to injection. Control and serine and glycine-free diets were obtained from IPS-TestDiet, formulations described previously [25].

### Targeted radiotherapy in vivo

Mice-bearing tumours were irradiated on an XStrahl Small Animal Radiation Research Platform (SARRP). Mice were anaesthetised with isoflurane and immobilised on a cradle with a tooth bar attachment. A Cone Beam Computed Tomography (CBCT) scan was performed before irradiation, using the parameters 60 kV and 0.8 mA. Integrated preclinical treatment planning software (Muiplan), was used to segment the tissues, select the isocentre and precisely target and plan the irradiation of the tumour. To ensure adequate coverage of the tumour, an appropriately sized collimator was used (10 mm by 10 mm, or motorised variable collimator (MVC) used at the required size), and the tumour was dosed using parallel opposed beams with 15 Gy to 20 Gy radiation, using the parameters 220 kV voltage and 13 mA current.

### Rectal cancer patient-derived tumour organoid (tumoroid) culture and irradiation

The rectal cancer patient-derived organoid biorepository at MSK is prospectively maintained by JJS and CW and is under the approved institutional review board protocol for JJS (#16-1071). All patients provided informed consent for use of tissue. For the derivation of tumoroids, fresh human rectal cancer samples were processed as reported [50] and culture and passage were as noted [51]. The basal culture medium for rectal cancer tumoroids was modified from the published method [50] as follows: Advanced DMEM/F12 (Gibco) was supplemented with antibiotic-antimycotic (Gibco), 1× B27 (Gibco), 1× N2 (Gibco), 2 mM GlutaMAX (Gibco), 10 nM human Gastrin I (Sigma-Aldrich), 10 mM HEPES (Gibco), 10 mM nicotinamide (Sigma-Aldrich), 50 ng ml^–1^ human recombinant human EGF (Peprotech), 500 nM A83-01 (Tocris Bioscience), and 10 μM SB 202190 (Sigma-Aldrich). For tumoroid nutrient modulation, cells were grown in the experimental medium using basal culture media described above but Advanced DMEM/F12 was substituted for Advanced DMEM/F12 media lacking serine and glycine (hereafter ‘-SG medium’) and matched media containing serine and glycine (hereafter ‘Control medium’).

For nutrient modulation and irradiation assays, 96-well plates were coated with 11% Matrigel (Corning, product no. 356231) in UltraPure Distilled Water (Invitrogen, product no. 10977), centrifuged at 300×*g* for 5 min, and were polymerised at least 30 min before seeding. Tumoroids were resuspended in 5% Matrigel in an experimental medium and embedded in suspension into coated well (5000–10,000 cells per 100 µl suspension per well), and centrifuged at 300×*g* for 5 min, and placed in an incubator to polymerise Matrigel. After Matrigel polymerised, 160 µl of culture medium was added per well after the Matrigel had polymerised. Radiation was delivered with an X-RAD320 Biological Irradiator (Precision X-Ray, North Branford, CT) set to deliver 4 Gy at 48 h post-seeding. Cells were grown in culture for a further 6 days post-irradiation with the media refreshed 2-days post radiation. At the end of treatment, the number of viable cells was determined by CellTiter-Glo assay (Promega), following the kit protocol.

### RNA extraction and RNA sequencing

The extraction and purification of total RNA was performed using the RNAeasy purification Kit (QIAGEN) combined with RNaseFree DNase (QIAGEN) treatment according to the manufacturer’s protocol. RNA concentration and quality was determined using the NanoDrop 2000 spectrophotometer before downstream processing. Quality of the purified RNA was tested on an Agilent 2200 Tapestation using RNA screentape. Libraries were prepared using the TruSeq stranded total RNA with RiboZero kit (Illumina). Quality and quantity of the cDNA libraries were assessed on an Agilent 2200 Tapestation (D1000 screentape). Libraries were run on an Illumina NextSeq500 using the High Output 75 cycles kit (2 × 36 cycles, paired-end reads, single index). Quality checks on the raw RNA-Seq data files were done using FastQC [52]. Alignment of the RNA-Seq paired-end reads was to the GRCm38 [53] version of the mouse genome and annotation using HiSat2 [54] and TopHat [55]. Gene expression levels were quantified using the feature Counts tool [56] available on Galaxy.org DESeq2 [57] was used to generate a list of differentially expressed protein-coding genes between. Calculations were performed with R version 3.3.1 in R-Studio (version 0.99.903). RNA-Seq data is available as GEO accession number GSE190668.

### Integrated pathway analysis of transcriptome and metabolomics

To integrate the metbolomics and gene expression data we used the “Integrated pathway analysis” tool of Metaboanalyst. Processed data were used for the analysis, specifically fold changes values for differentially expressed genes or metabolites for the relevant comparison were used as input for the integrated analysis (https://www.metaboanalyst.ca/home.xhtml; [58], based on the work of Cavill et al. [59].

### LCMS for steady-state metabolite measurements

Metabolomics experiments were performed as described previously [25, 49]. Cells were seeded into six-well plates in complete medium and allowed to attach overnight. Cells were washed with PBS, and the relevant experimental media change was performed followed by irradiation. Cells were then cultured for the stated amount of time. Duplicate wells were used for cell counting to normalise the volume of lysis solvent prior to metabolite extractions (2 × 10^6^ cells per ml). Briefly, cells were washed quickly in PBS, then an ice-cold lysis solvent (methanol 50%, acetonitrile 30%, water 20%) was added, and cells were scraped on ice. Lysates were transferred to 1.5-ml tubes on ice, vortexed, then centrifuged at 18,000×*g* at 4 °C for 10 min. Supernatants were collected and stored at 80 °C for LCMS analysis. Tissue samples were snap-frozen and stored at 80 °C. Frozen samples were weighed before lysis. Samples were homogenised in 2 ml ice-cold lysis solvent using a TissueLyser II (QIAGEN). Lysates were then cleared of protein by centrifugation at 18,000×*g* for 10 mins at 4 °C and then normalised to 10 mg/ mL with lysis buffer based on original tissue mass. Sample analysis was performed using an LCMS platform consisting of an Accela 600 LC system and an Exactive mass spectrometer (Thermo Scientific). A Sequant ZIC-pHILIC column (4.6 mm × 150 mm, 3.5 mm) (Merck) was used to separate the metabolites with the mobile phase mixed by A = 20 mM ammonium carbonate in water and B = acetonitrile. A gradient programme starting at 20% of A and linearly increasing to 80% at 30 min was used followed by washing (92% of A for 5 min) and re-equilibration (20% of A for 10 min) steps. The total run time of the method was 45 min. The LC stream was desolvated and ionised in the HESI probe. The Exactive mass spectrometer was operated in full scan mode over a mass range of 70–1200 *m/z* at a resolution of 50,000 with polarity switching. The LCMS raw data were converted into mzML files by using the MSConvert tool (ProteoWizard) and imported to MZMine 2.3 for peak picking and sample alignment. A house-made database including all possible 13 C and 15 N isotopic *m/z* values of the relevant metabolites was used for the assignment of LCMS signals. Finally, the peak areas were used for comparative quantification.

### Unbiased metabolomics

Metabolomic statistical analyses were done in MetaboAnalyst 4.0 using metabolite peak areas. Principal component analysis (PCA) and partial least squares-discriminant analysis (PLS-DA) were performed using log_10_-transformed data. PLS-DA was used for variable selection by ranking the variable importance in decreasing order. The pathway analysis and enrichment analysis modules in MetaboAnalyst 4.0 were used to match the metabolites with their corresponding pathways.

### Quantification and statistical analysis

The number of biological replicates per experiment and the number of experiments performed for each data set and the statistical analysis performed are stated in the figure legends. Results are depicted as mean ±  standard deviation (SD) unless otherwise stated using Microsoft Excel for Mac (v16.56) or GraphPad Prism (v.9.2.0) or R version 3.3.1 in R-Studio (version 0.99.903). *P* values were calculated either using GraphPad Prism (v.9.2.0) or Microsoft Excel for Mac (v16.55). No statistical method was used to predetermine sample size. Sample sizes were estimated according to common practice for each experimental design and based on previous experience and pilot experiments to estimate variability.

## Supplementary information

Supplementary Figures

Checklist

## Data Availability

Source data are made available at https://researchdata.gla.ac.uk.
