# Supplementary material for: Sensitisation of cancer cells to radiotherapy by serine and glycine starvation
Source: Br J Cancer. 2022 Sep 17;127(10):1773–86. doi: 10.1038/s41416-022-01965-6 (PMC9643498; doi:10.1038/s41416-022-01965-6)
Supplement: Supplementary file 1 — Supplementary Figures [file 41416_2022_1965_MOESM1_ESM.pdf]

Falcone et. al.,
Supplementary Information

Figure S1

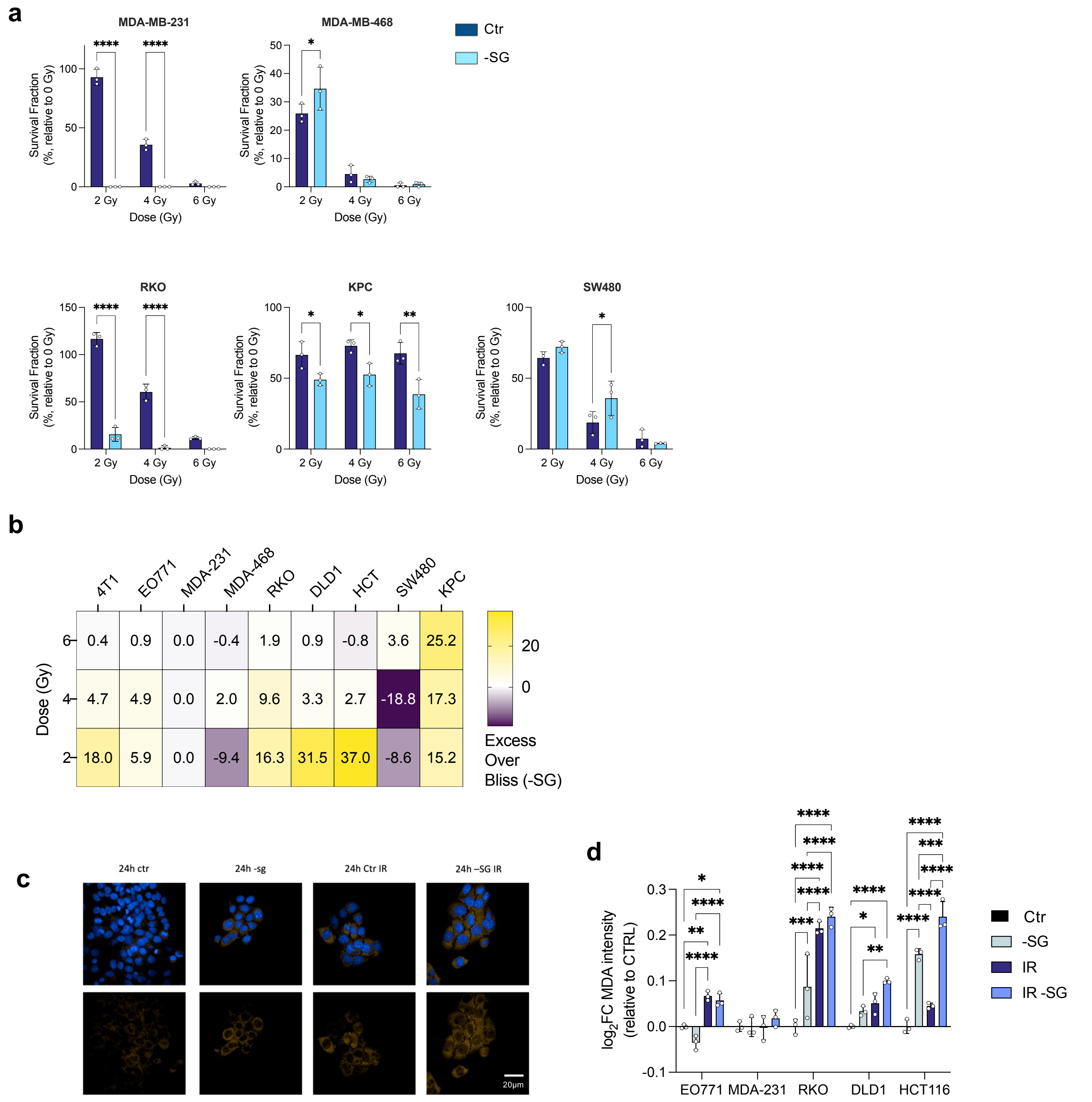

Figure S1. Serine and glycine starvation sensitises a range of cancer cells to radiation *in vitro* (linked to Figure 1)

a) Quantification of clonogenic assays for the displayed cell lines cultured with media containing serine and glycine (+SG) or not (-SG) and irradiated with increasing dose of radiation (0-6 Gy); surviving fraction relative to non-irradiated controls (mean +/-SD, one experiment in biological triplicate, Two-way ANOVA with Šídák's multiple comparisons test, $*p<0.05$, $**p<0.005$, $***p<0.0005$, $****p<0.00005$). For MDA-MB-231 cells there was complete cell death in the –SG condition for all doses of radiation.

b) Excess over Bliss score based on clonogenic assays of displayed lines cultured with media containing serine and glycine or not and irradiated with increasing dose of radiation (0-6 Gy) (mean, n = 4 experiments in biological triplicate for 4T1 and one experiment in biological triplicate for other cell lines).

c) Representative images of malondialdehyde (MDA) adduct detected using immunofluorescence staining 24h after treatment. 4T1 cells were cultured with media containing serine and glycine (Ctr) or not (-SG) and irradiated with a final dose of 4Gy X-ray radiation (IR) (scale bar 20um).

d) Quantification of malondialdehyde (MDA) adduct intensity relative to Ctr measured using immunofluorescence staining 72h after treatment. Cells were cultured with media containing serine and glycine (+SG) or not (-SG) and irradiated with a final dose of radiations of 4Gy (IR). (mean +/-SD, one experiment in biological triplicate, Two-way ANOVA with Tukey's multiple comparisons test, $*p<0.05$, $**p<0.005$, $***p<0.0005$, $****p<0.00005$).

Figure S2

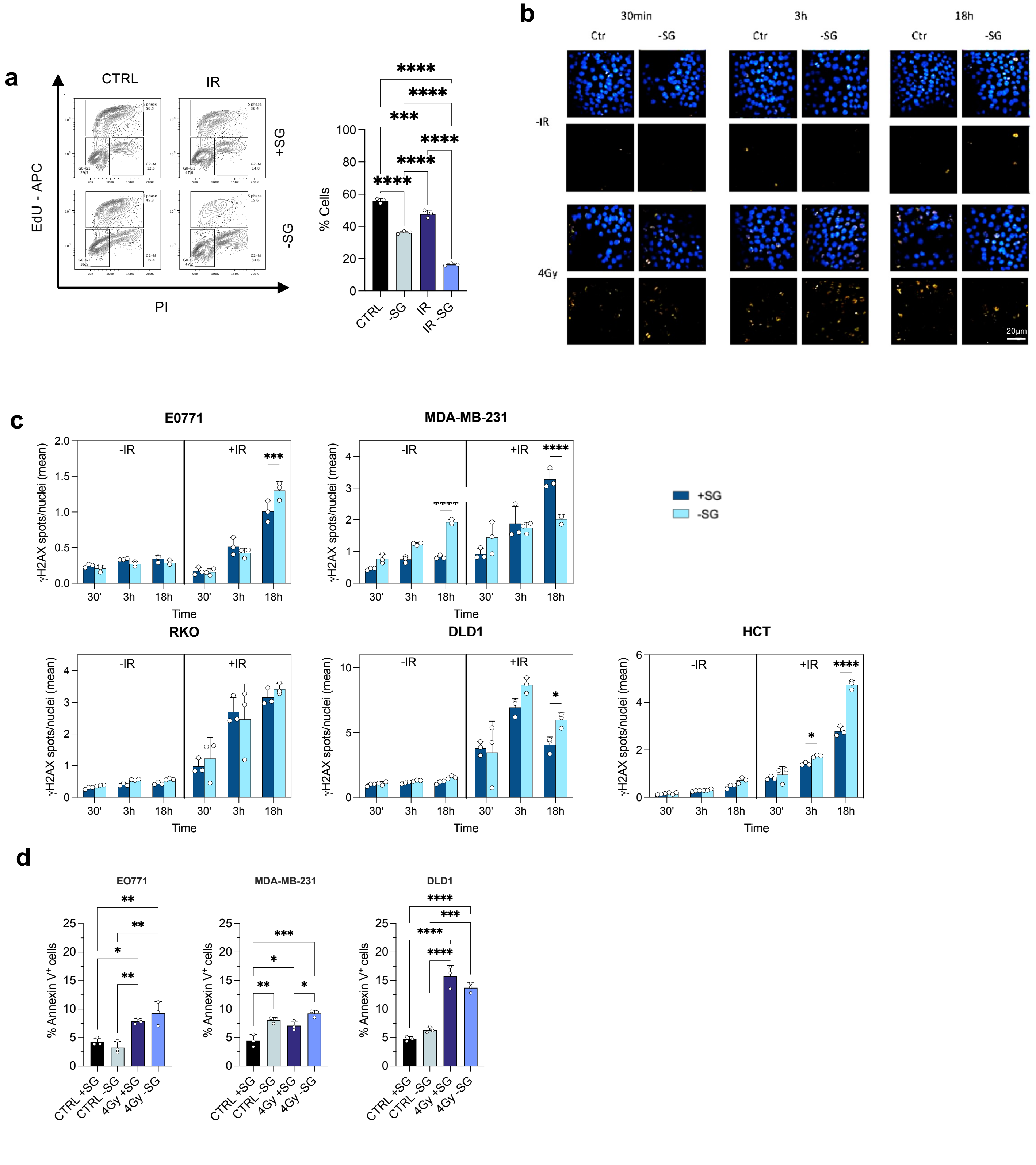

Figure S2. Serine and glycine starvation increases damage from radiation *in vitro* (linked to Figure 1)

a) EdU incorporation measured using flow cytometry 48h after treatment. Representative FACS plots of EdU fluorescence vs. Propidium Iodide (PI) of HCT116 cells cultured with media containing serine and glycine (+SG; top row) or not (-SG; bottom row) and irradiated with a final dose of radiations of 4Gy (IR). Numbers indicate percentage of EdU positive cells in each gate, with quantification (mean +/-SD, one experiment in biological triplicate, One-way ANOVA with Tukey's multiple comparisons test, *p<0.05, **p<0.005, ***p<0.0005, ****p<0.00005).

b) Representative images of γ-H2AX foci at different time points using immunofluorescence staining in 4T1 cells cultured with media containing serine and glycine (Ctr) or not (-SG) and irradiated or not with a final dose of 4Gy X-ray radiation (+/-IR) (scale bar 20um).

c) Quantification of γ-H2AX foci at different time points in cells cultured with media containing serine and glycine (+SG) or not (-SG) and irradiated with a final dose of radiations of 4Gy (IR) (mean +/-SD, one experiment in biological triplicate, Two-way ANOVA with Tukey's multiple comparisons test, *p<0.05, **p<0.005, ***p<0.0005, ****p<0.00005).

d) Quantification of Annexin V positive cells using flow cytometry 24h after treatment. Cells cultured with media containing serine and glycine (+SG; top row) or not (-SG; bottom row) and irradiated with a final dose of radiations of 4Gy (IR). Numbers indicate percentage of positive cells (mean +/-SD, one experiment in biological triplicate, One-way ANOVA with Tukey's multiple comparisons test, *p<0.05, **p<0.005, ***p<0.0005, ****p<0.00005).

Figure S3

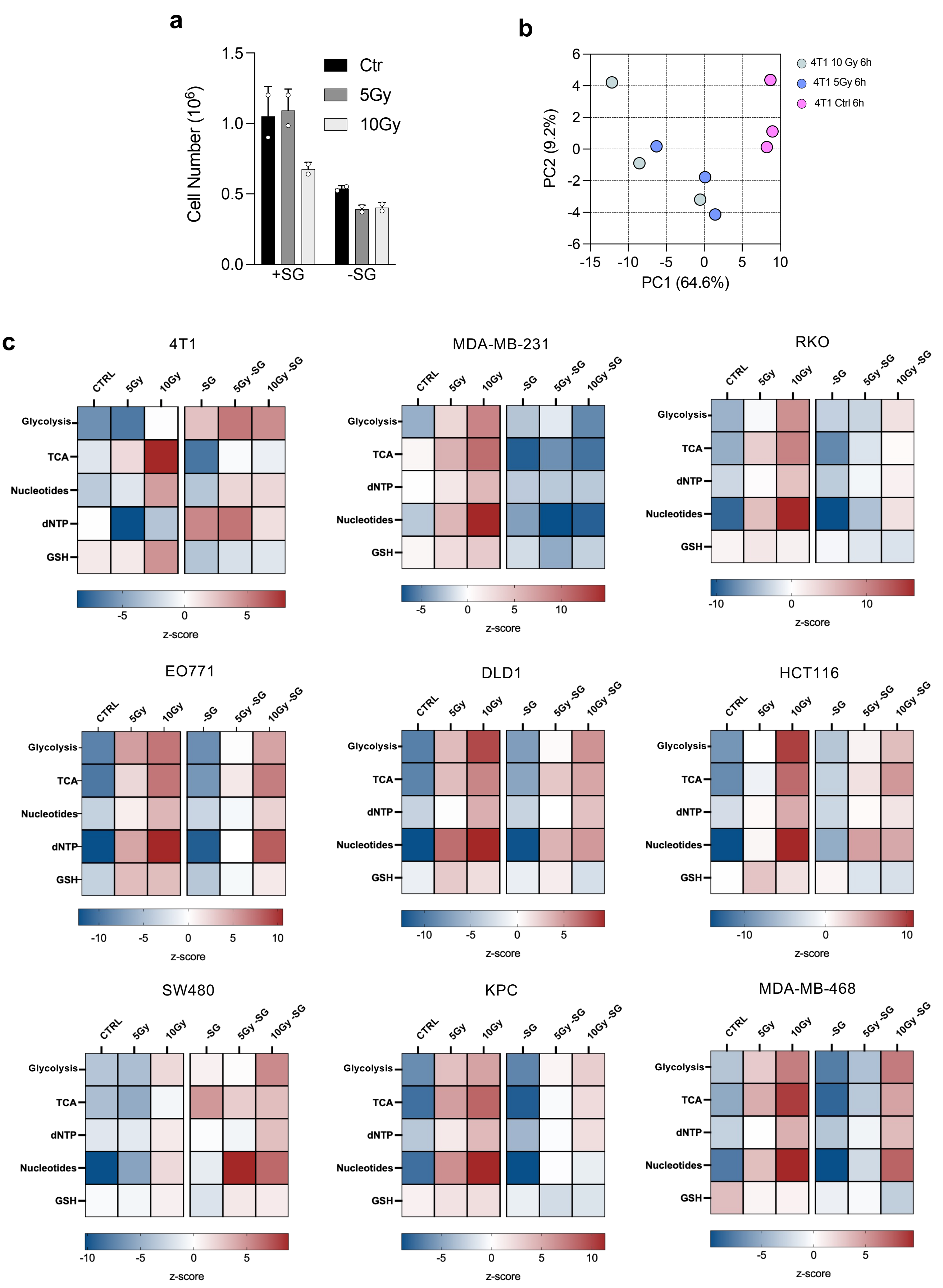

Figure S3. Metabolomic impact of radiation and SG starvation (linked to Figure 2)

a) Absolute number of 4T1 cells used for metabolomics experiment (shown in Figure 3) cultured with media containing serine and glycine (Ctr) or not (-SG) and irradiated with increasing doses of X-ray radiation (0, 5 and 10 Gy) for 24h (mean +/-SD, counts done in a single experiment in biological duplicate).

b) Spatial division displayed as PCA plot based on the metabolomic profile of 4T1 cells cultured in complete medium irradiated with increasing dose of X-ray radiation (0/Ctr, 5 and 10 Gy) and harvested at 6h post-radiation (one experiment in biological triplicate).

c) Summarized pathway activity for the displayed cell lines cultured with media containing serine and glycine (CTRL) or not (-SG) and irradiated with increasing dose of radiations (0, 5 and 10 Gy) for 24h (one experiment in biological triplicate for each cell line).

Figure S4

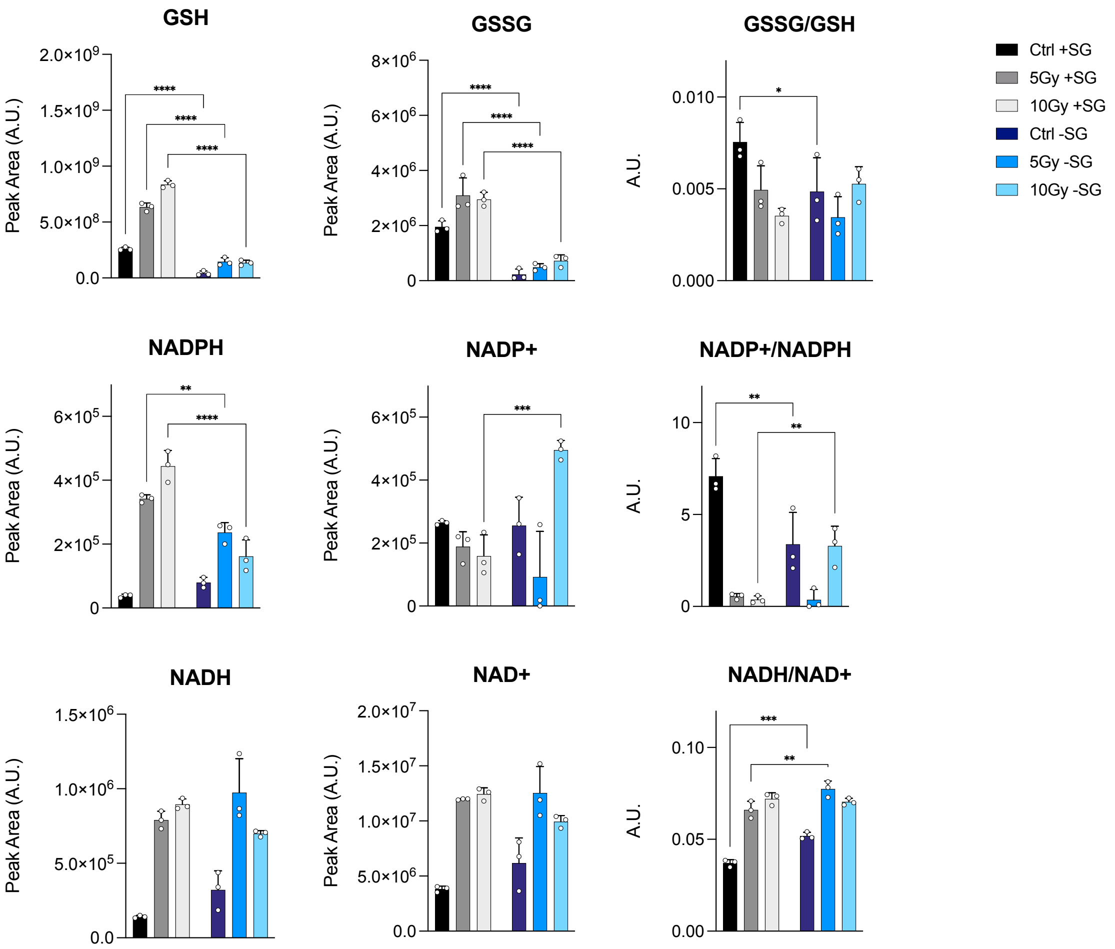

Figure S4. Impact of radiotherapy on redox related metabolites (linked to Figure 3)
Metabolite levels analysed by LCMS in 4T1 cells cultured with media containing serine and glycine (+SG) or not (-SG) and irradiated with increasing doses of X-ray radiation (0, 5 and 10 Gy) for 24h (mean +/-SD, one experiment in biological triplicate, Two-way ANOVA with Šídák's multiple comparisons test, *p<0.05, **p<0.005, ***p<0.0005, ****p<0.00005).

Figure S5

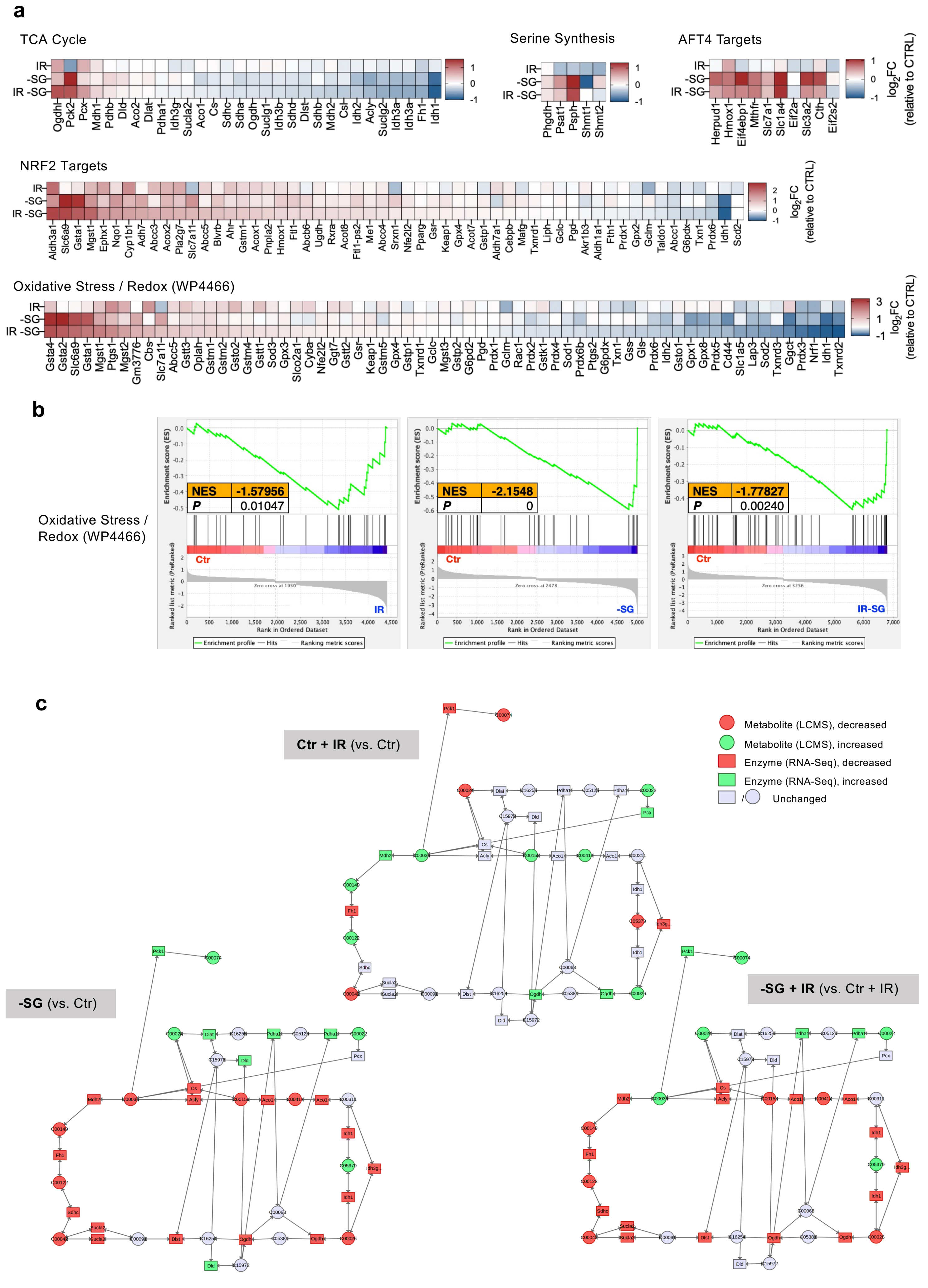
a
TCA Cycle
IR
-SG
IR -SG
Ogdhl Pck2 Pcx Mdh1 Pdhb Dld Aco2 Dlat Pdha1 Idh3g Sucla2 Aco1 Cs Sdhc Sdha Ogdh Suclg1 Idh3b Sdhd Dlst Sdhb Mdh2 Csl Idh2 Acly Suclg2 Idh3a Idh3a Fh1 Idh1
Serine Synthesis
Phgdh Psat1 Psph Pgd Shmt1 Shmt2
AFT4 Targets
Herpud1 Hmox1 Eif4ebp1 Mthfr Slc7a11 Slc1a4 Eif2a Slc3a2 Cth Eif2s2
log2FC (relative to CTRL)
NRF2 Targets
Aldh3a1 Slc6a9 Gsta1 Mgst1 Ephx1 Nqo1 Cyp1b1 Adh7 Abcc3 Acox2 Pla2g7 Slc7a11 Abcc5 Blvrb Ahr Gstm1 Acox1 Pnpla2 Hmox1 Ftl Abcb6 Ugdh Rxra Acot8 Ftl1-ps2 Me1 Abcc4 Srxn1 Nfe2l2 Pparg Gsr Keap1 Gpx4 Acot7 Gstp1 Aldh7a1 Cebpb Mafg Txnrd1 Liph Gclc Pgd Akr1b3 Aldh1a1 Fth1 Prdx1 Gpx2 Gclm Taldo1 Abcc1 G6pdx Txn1 Prdx6 Idh1 Scd2
Oxidative Stress / Redox (WP4466)
Gsta4 Gsta2 Slc6a9 Gsta1 Mgst1 Ptgs1 Mgst2 Gm3776 Cbs Slc7a11 Abcc5 Gstt3 Oplah Gstm1 Gstm2 Gsto2 Gstm4 Gstt1 Sod3 Gpx3 Slco2a1 Cyba Nfe2l2 Ggt7 Gstt2 Gsr Keap1 Gstm5 Gpx4 Gstp1 Txnrd1 Gclc Mgst3 Gstp2 G6pd2 Pgd Gclm Rac1 Prdx2 Gstk1 Prdx4 Sod1 Prdx6b Ptgs2 G6pdx Txn1 Gss Gls Prdx6 Idh2 Gsto1 Gpx1 Gpx8 Prdx5 Cd44 Slc1a5 Lap3 Sod2 Txnrd3 Ggct Prdx3 Nrf1 Idh1 Txnrd2
b
Oxidative Stress / Redox (WP4466)
NES -1.57956
P 0.01047
Ctr
IR
Zero cross at 1950
NES -2.1548
P 0
Ctr
-SG
Zero cross at 2478
NES -1.77827
P 0.00240
Ctr
IR-SG
Zero cross at 3256
Enrichment score (ES)
Ranked list metric (PreRanked)
Rank in Ordered Dataset
Enrichment profile — Hits Ranking metric scores
c
Ctr + IR (vs. Ctr)
-SG (vs. Ctr)
-SG + IR (vs. Ctr + IR)
Metabolite (LCMS), decreased
Metabolite (LCMS), increased
Enzyme (RNA-Seq), decreased
Enzyme (RNA-Seq), increased
Unchanged

Figure S5

Figure S5. TCA cycle ans stress responses impact of radiation and SG starvation (linked to Figure 4)

a) Heat map representation of gene expression (as log_2FC relative to Ctr condition) of members of the displayed pathways based on RNA-Seq profile of 4T1 cells cultured with media containing either serine and glycine (+SG) or not (-SG) and subsequently irradiated with a final dose of radiations 5 Gy (IR) for 24h.

b) GSEA of the IR, -SG, IR-SG vs Ctr respectively using the WP4466 (Oxidative stress / Redox) gene sets (NES, normalized enrichment score).

c) TCA cycle KEGG pathway overview displaying both enzyme (rectangles) and metabolite (circles) ratios between the highlighted conditions (one experiment in biological triplicate for both metabolomics profiling and RNA-Sequencing).

Figure S6

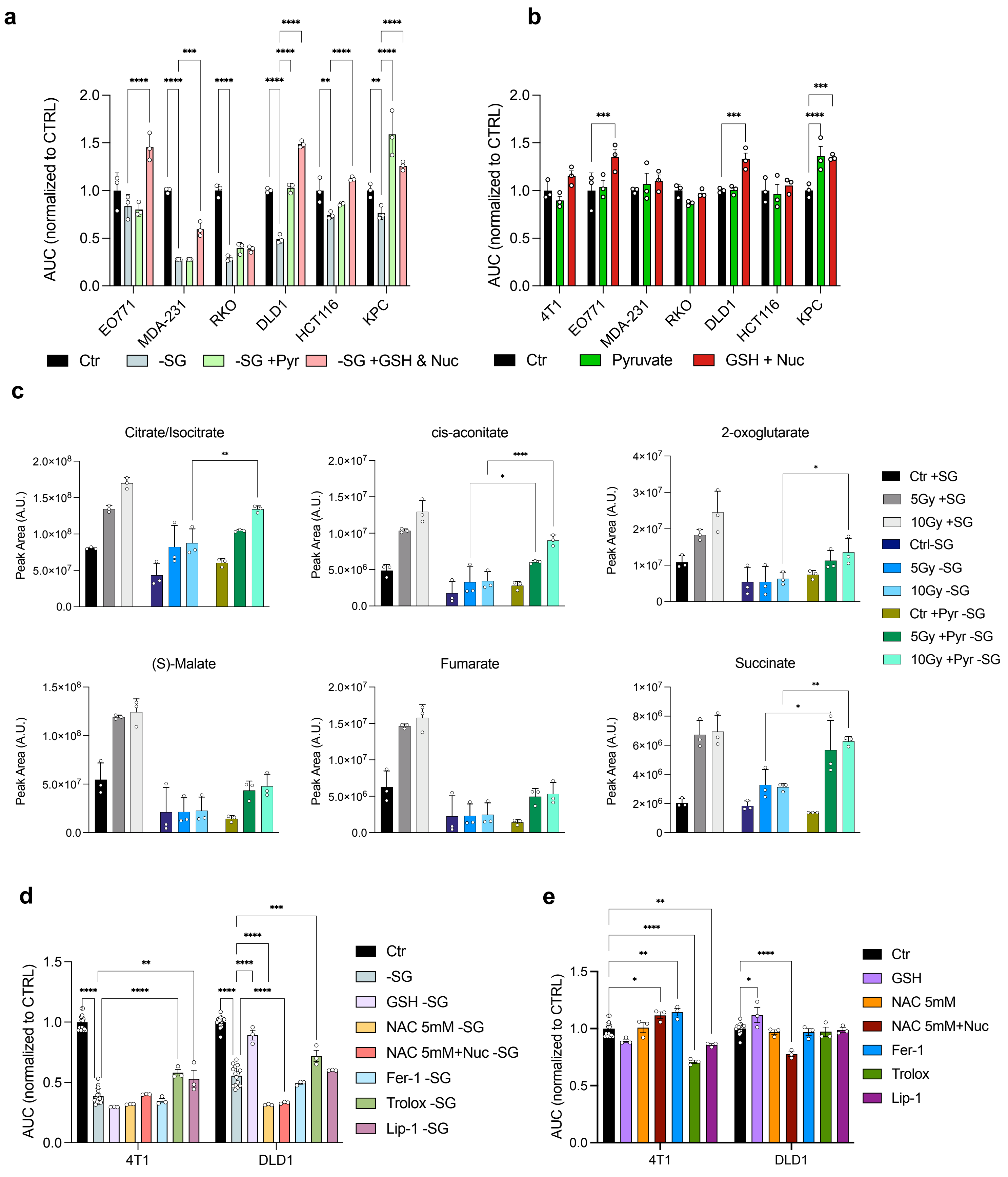

a
AUC (normalized to CTRL)
EO771
MDA-231
RKO
DLD1
HCT116
KPC
Ctr
-SG
-SG +Pyr
-SG +GSH & Nuc
b
AUC (normalized to CTRL)
4T1
EO771
MDA-231
RKO
DLD1
HCT116
KPC
Ctr
Pyruvate
GSH + Nuc
c
Citrate/Isocitrate
cis-aconitate
2-oxoglutarate
(S)-Malate
Fumarate
Succinate
Peak Area (A.U.)
Ctr +SG
5Gy +SG
10Gy +SG
Ctrl-SG
5Gy -SG
10Gy -SG
Ctr +Pyr -SG
5Gy +Pyr -SG
10Gy +Pyr -SG
d
AUC (normalized to CTRL)
4T1
DLD1
Ctr
-SG
GSH -SG
NAC 5mM -SG
NAC 5mM+Nuc -SG
Fer-1 -SG
Trolox -SG
Lip-1 -SG
e
AUC (normalized to CTRL)
4T1
DLD1
Ctr
GSH
NAC 5mM
NAC 5mM+Nuc
Fer-1
Trolox
Lip-1

Figure S6

Figure S6. Metabolite rescue (linked to Figure 4)

a) Clonogenic assay shown as normalized area under the curve (AUC) of the displayed cells lines cultured with media containing serine and glycine (Ctr) or not (-SG), irradiated with increasing dose of X-ray radiation (0-6 Gy) and with or without supplementation with 2mM Pyruvate or 2.5mM reduced glutathione & nucleosides mix (mean +/-SD, one experiment in biological triplicate, Two-way ANOVA with Dunnet's multiple comparisons test, $^{*}p<0.05$, $^{**}p<0.005$, $^{***}p<0.0005$, $^{****}p<0.00005$).

b) Clonogenic assay shown as normalized area under the curve (AUC) of the displayed cells lines cultured with media containing serine and glycine, irradiated with increasing dose of radiations (0-6 Gy) and supplemented with either 2mM Pyruvate or with 2.5mM reduced glutathione & nucleosides mix (mean +/-SD, one experiment in biological triplicate, Two-way ANOVA with Dunnet's multiple comparisons test, $^{*}p<0.05$, $^{**}p<0.005$, $^{***}p<0.0005$, $^{****}p<0.00005$).

c) Metabolite levels analysed by LCMS in 4T1 cells cultured with media containing serine and glycine (Ctr) or not (-SG) and with or without 2mM Pyruvate, subsequently irradiated with increasing doses of X-ray radiation (0, 5 and 10 Gy) for 24h (mean +/-SD, one experiment in biological triplicate Two-way ANOVA with Tukey's multiple comparisons test, $^{*}p<0.05$, $^{**}p<0.005$, $^{***}p<0.0005$, $^{****}p<0.00005$).

d) Clonogenic assay shown as normalized area under the curve (AUC) of 4T1 and DLD1 lines cultured with media containing serine and glycine (Ctr) or not (-SG), subsequently irradiated with increasing dose of radiations (0-6 Gy) and supplemented with 2.5mM reduced glutathione (GSH), 5mM N-acetylcysteine (NAC), 5mM NAC+nucleosides mix (NAC+Nuc), 2uM Ferrostatin-1 (Fer-1), 50uM Trolox or 100nM Liproxstatin-1 (Lip-1) (mean +/-SD, one experiment in biological triplicate, Two-way ANOVA with Dunnet's multiple comparisons test, $^{*}p<0.05$, $^{**}p<0.005$, $^{***}p<0.0005$, $^{****}p<0.00005$).

e) Clonogenic assay shown as normalized area under the curve (AUC) of 4T1 and DLD1 lines cultured with media containing serine and glycine, subsequently irradiated with increasing dose of radiations (0-6 Gy) and supplemented with 2.5mM reduced glutathione (GSH), 5mM N-acetylcysteine (NAC), 5mM NAC+nucleosides mix (NAC+Nuc), 2uM Ferrostatin-1 (Fer-1), 50uM Trolox or 100nM Liproxstatin-1 (Lip-1) (mean +/-SD, one experiment in biological triplicate, Two-way ANOVA with Dunnet's multiple comparisons test, $^{*}p<0.05$, $^{**}p<0.005$, $^{***}p<0.0005$, $^{****}p<0.00005$).

Figure S7

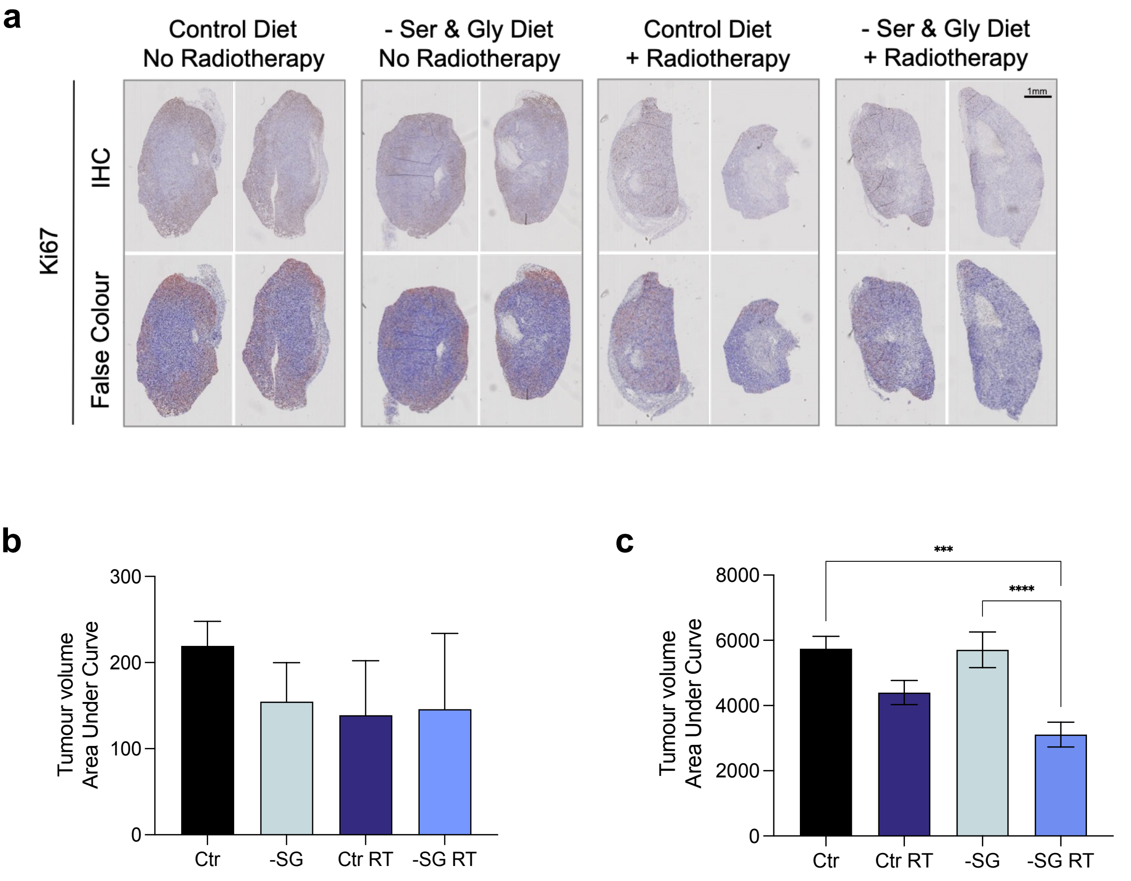

Figure S7. Tumour growth area under curve (linked to figure 5)

a) Representative formalin fixed paraffin embedded tissue sections from KPC cell syngeneic subcutaneous isografts were stained for Ki67. Once measurable tumours had formed, mice were transferred to a diet regime consisting of either a control diet containing serine and glycine (Control diet) or a matched diet lacking serine and glycine (-SG diet); after three days on diet, tumours were treated with 20 Gy targeted X-ray radiation. None of the tumours reached size endpoint due to skin ulceration occurring in all experimental groups, so all tumours were harvested for IHC analysis within 7 days of radiation.

b) Quantification of KPC cell syngeneic subcutaneous isografts shown in Fig. 5a as Area under the curve (AUC) for tumour volume (mean +/-SD, Control diet No RT n = 2 mice, Control diet +RT *n* =4 mice, -SG diet No RT *n* = 6 mice, -SG diet +RT *n* = 6 mice).

c) Quantification of Fig. 5c. 4T1 cell orthotopic isografts as Area under the curve (AUC) for tumour volume (mean +/-SD, Control diet No RT n = 13 mice, Control diet +RT *n* =12 mice, -SG diet No RT *n* = 15 mice, -SG diet +RT *n* = 12 mice, Two-way ANOVA with Tukey's multiple comparisons test, *p<0.05, **p<0.005, ***p<0.0005, ****p<0.00005).

	Ctr vs 5Gy	Ctr vs 10Gy	Ctr vs -SG	Ctr vs 5Gy -SG	Ctr vs 10Gy -SG	5Gy vs 10Gy	5Gy vs -SG	5Gy vs 5Gy -SG	5Gy vs 10Gy -SG	10Gy vs -SG	10Gy vs 5Gy -SG	10Gy vs 10Gy -SG	5Gy -SG vs 10Gy -SG	-SG vs 5Gy -SG	-SG vc 10Gy -SG
D-Glucose	0.4603	0.9802	0.0264	0.1608	0.0223	0.8346	0.4654	0.9685	0.4139	0.0833	0.4191	0.0707	0.8299	0.8729	>0.9999
Fructose 1,6-bisphosphate	0.2032	0.6333	0.0017	0.7586	0.2835	0.9341	<0.0001	0.0228	0.0050	0.0002	0.1054	0.0231	0.9360	0.0152	0.0707
Glyceraldhyde 3-phosphate	0.0074	0.3066	0.0082	<0.0001	0.0001	0.2654	<0.0001	<0.0001	<0.0001	0.0003	<0.0001	<0.0001	0.7110	0.0116	0.1256
1,3-bisphosphoglycerate	0.9957	0.0027	0.0021	0.0002	0.0004	0.0012	0.0010	<0.0001	0.0002	>0.9999	0.5154	0.7905	0.9955	0.6019	0.8613
3-phosphoglycerate	0.9957	0.0997	0.1862	0.0099	0.2070	0.2130	0.3695	0.0225	0.4038	0.9983	0.7378	0.9964	0.4735	0.5121	>0.9999
PEP	>0.9999	0.0393	0.0047	0.0013	0.0199	0.0392	0.0047	0.0013	0.0198	0.7941	0.3621	0.9982	0.5760	0.9625	0.9474
Pyruvate	0.9118	0.0499	0.9855	0.0315	0.0032	0.2411	0.9991	0.1600	0.0167	0.1415	0.9997	0.5887	0.7437	0.0912	0.0092
Acetyl-CoA	0.8776	>0.9999	<0.0001	<0.0001	<0.0001	0.9090	<0.0001	<0.0001	<0.0001	<0.0001	<0.0001	<0.0001	>0.9999	0.0034	0.0045
Citrate/Isocitrate	0.7060	0.0027	0.4233	0.8940	0.9628	0.0294	0.0481	0.9984	0.9853	0.0001	0.0151	0.0100	0.9998	0.0923	0.1372
cis-Aconitate	0.2264	0.0005	0.5821	0.9915	>0.9999	0.0216	0.0152	0.4761	0.2107	<0.0001	0.0011	0.0004	0.9877	0.2961	0.6104
2-Oxoglutarate	0.5011	0.0345	0.2279	0.9778	0.9363	0.5105	0.0120	0.1978	0.1442	0.0007	0.0105	0.0075	>0.9999	0.5549	0.6733
Succinate	0.9955	>0.9999	0.6000	0.9997	0.6695	0.9787	0.8596	>0.9999	0.9061	0.4846	0.9956	0.5522	0.8215	0.7607	>0.9999
Fumarate	0.6708	0.0005	0.5466	0.9895	0.9803	0.0047	0.0634	0.9378	0.9591	<0.0001	0.0011	0.0013	>0.9999	0.2612	0.2293
Malate	0.6828	0.0009	0.4274	0.9896	0.9582	0.0093	0.0455	0.9427	0.9833	<0.0001	0.0022	0.0031	0.9999	0.1901	0.1339
Oxaloacetate	0.9982	0.5834	0.1714	0.3098	0.8540	0.3671	0.0900	0.1720	0.6418	0.9267	0.9934	0.9948	0.8938	0.9981	0.6973
dATP	0.0724	<0.0001	0.1864	0.0269	0.0306	<0.0001	0.0011	0.9897	0.9945	<0.0001	0.0001	<0.0001	>0.9999	0.0005	0.0005
dGTP	0.0028	0.0023	0.1810	0.1069	0.8815	>0.9999	<0.0001	0.3048	0.0005	<0.0001	0.2561	0.0004	0.0177	0.0016	0.6796
dCTP	0.9569	0.0002	0.9997	0.0006	0.0009	0.0007	0.9931	0.0021	0.0033	0.0003	0.9836	0.9258	0.9996	0.0009	0.0014
dTTP	0.2222	<0.0001	0.8165	<0.0001	<0.0001	<0.0001	0.8300	0.0002	0.0001	<0.0001	0.3835	0.5215	0.9998	<0.0001	<0.0001
Serine	0.8875	<0.0001	<0.0001	<0.0001	<0.0001	0.0004	<0.0001	<0.0001	<0.0001	<0.0001	<0.0001	<0.0001	>0.9999	>0.9999	>0.9999
Glycine	<0.0001	0.0078	<0.0001	<0.0001	<0.0001	<0.0001	<0.0001	<0.0001	<0.0001	<0.0001	<0.0001	<0.0001	0.9798	0.9915	0.8021

Table S1. P-values from two-way ANOVA with Tukey's multiple comparison tests ($p < 0.05$ are shaded grey) for the LCMS data shown in Figure 3.
